# Data-Driven Engineering of Antimicrobial Nanomaterials for Food Safety and Biomedical Systems

**DOI:** 10.3390/nano16120764

**Published:** 2026-06-17

**Authors:** Huy Loc Nguyen, Hong Minh Xuan Nguyen, Thi Bich Ngoc Nguyen

**Affiliations:** 1 Department of Engineering and Technology, Van Hien University, Ho Chi Minh City 72419, Vietnam; 2 Department of Chemical Engineering and Food Technology, Nong Lam University, Ho Chi Minh City 71308, Vietnam; nmxhong@hcmuaf.edu.vn; 3 Department of Water Management and Hydrological Science, Texas A&M University, 3147 Tamu, College Station, TX 77843, USA

**Keywords:** antimicrobial, biomedical, data-driven design, food safety, nanomaterials, machine learning

## Abstract

Antimicrobial resistance and biofilm-associated contamination continue to pose critical challenges in food safety and biomedical applications, necessitating the development of advanced antimicrobial materials with enhanced efficacy, safety, and functional adaptability. Antimicrobial nanomaterials offer versatile solutions due to their tunable physicochemical properties, surface engineering capabilities, and controlled release behaviors, enabling improved antimicrobial and antibiofilm performance across diverse systems. This review highlights the main advancements in AI-assisted design of antimicrobial nanomaterials, demonstrating how data-driven approaches are increasingly used to predict antimicrobial activity, optimize synthesis parameters, model nanotoxicity, integrate multimodal datasets, and improve interpretability through explainable AI frameworks. Key findings indicate that machine learning-guided strategies and autonomous experimental platforms significantly accelerate material optimization while reducing reliance on traditional trial-and-error methods. The review further summarizes the performance and mechanisms of major antimicrobial nanomaterial systems, including metal and metal oxide nanoparticles, metal–organic frameworks, polymeric nanocarriers, nanoemulsions, and hybrid nanostructures, with emphasis on their translational applications in food preservation, antimicrobial coatings, wound healing, implant protection, and drug delivery. Despite these advances, challenges remain in data quality, model generalizability, toxicity prediction, reproducibility, and regulatory translation. AI-enabled and data-driven frameworks provide a powerful pathway for accelerating the rational design and practical implementation of next-generation antimicrobial nanomaterials.

## 1. Introduction

The rapid emergence of antimicrobial resistance (AMR) and the persistence of microbial biofilms in food and biomedical environments have become major global concerns, posing substantial threats to public health, food security, and healthcare systems worldwide [1]. The development of resistant microbial strains is increasingly limiting conventional antimicrobial strategies, reducing therapeutic efficacy and leading to undesirable toxicological effects [2]. Antimicrobial resistance is estimated to contribute to millions of deaths annually and is projected to impose substantial economic and healthcare burdens worldwide, highlighting the urgent need for innovative antimicrobial strategies. In this context, nanomaterials have attracted considerable attention for their unique physicochemical characteristics, including high surface-area-to-volume ratios, tunable surface chemistry, enhanced reactivity, and multifunctional antimicrobial mechanisms [3]. These properties enable nanomaterials to interact effectively with microbial membranes, induce oxidative stress, disrupt intracellular pathways, and inhibit biofilm formation.

Over the past decade, diverse classes of antimicrobial nanomaterials, including metal and metal oxide nanoparticles, metal–organic frameworks (MOFs), polymeric nanocarriers, and nanoemulsions, have been extensively investigated for applications in food preservation, surface decontamination, wound healing, drug delivery, and infection control [4,5]. Nevertheless, the rational development of these systems remains challenging due to the complex interplay between nanomaterial composition, morphology, surface properties, and biological performance. Minor variations in nanoparticle size, crystallinity, surface functionalization, or dopant incorporation may substantially alter antimicrobial efficacy, biocompatibility, and environmental behavior [6]. This high-dimensional design space complicates the identification of optimal nanomaterial configurations with conventional experimental approaches, necessitating predictive, data-driven design frameworks. Despite significant progress, many studies continue to rely heavily on empirical trial-and-error methodologies, which are often time-consuming, resource-intensive, and insufficient for elucidating comprehensive structure–function relationships.

Recent advances in artificial intelligence (AI), machine learning (ML), and materials informatics have introduced transformative opportunities for the rational design and optimization of functional nanomaterials [7]. Data-driven approaches enable the analysis of large and multidimensional datasets to identify hidden correlations between physicochemical descriptors and functional performance. In materials science, ML algorithms have demonstrated remarkable capability in accelerating materials discovery, predicting material properties, optimizing synthesis parameters, and guiding inverse design strategies [7,8]. Furthermore, the emergence of high-throughput computational frameworks and open-access materials databases has significantly enhanced the integration of experimental and computational workflows for materials development [9].

Within antimicrobial nanotechnology, data-driven methodologies are increasingly being employed to predict antibacterial activity, evaluate cytotoxicity, optimize formulation parameters, and advance mechanistic understanding of nano–bio interactions [10]. Predictive models grounded in physicochemical descriptors, spectroscopic datasets, and quantitative imaging analyses have demonstrated considerable potential for reducing experimental burden while enhancing design efficiency [11]. The integration of AI with automated and closed-loop experimental platforms has further accelerated the emergence of autonomous materials discovery systems capable of executing iterative cycles of synthesis, characterization, and performance optimization [12]. Such approaches are especially promising for antimicrobial nanomaterials, where the high dimensionality of experimental parameter spaces frequently confounds conventional optimization strategies.

Notwithstanding these advances, several fundamental limitations remain unresolved. The absence of standardized, high-quality training datasets, combined with constraints on model interpretability, insufficient external validation, and persistent reproducibility challenges, continues to impede the broad implementation of AI-driven nanomaterial design [13]. Compounding these issues, the inherent biological complexity of food and biomedical matrices introduces substantial experimental variability that may restrict model transferability across disparate application contexts and environmental conditions [14]. Overcoming these barriers necessitates substantive interdisciplinary integration across nanotechnology, microbiology, computational science, and data engineering to establish predictive frameworks of sufficient rigor and generalizability. Although recent reviews have discussed antimicrobial nanomaterials, materials informatics, or artificial intelligence in materials science independently, a comprehensive synthesis specifically addressing AI-assisted design strategies for antimicrobial nanomaterials across food safety and biomedical applications remains limited. In particular, the integration of descriptor-based modeling, machine learning-guided synthesis optimization, explainable AI, autonomous experimentation, and translational challenges has not been systematically evaluated within a unified framework.

Against this backdrop, this work provides a comprehensive and critical synthesis of recent advances in AI-enabled antimicrobial nanomaterial design for food safety and biomedical applications. Unlike previous reviews that primarily focus on either nanomaterial platforms or computational methodologies separately, this work integrates antimicrobial mechanisms, descriptor-based prediction, machine learning-guided synthesis, autonomous experimentation, nanotoxicity assessment, and translational challenges within a unified data-driven framework. By identifying current limitations and emerging opportunities, this work aims to establish practical design principles for the rational development of next-generation antimicrobial nanomaterials.

## 2. Methodology

This work was conducted through a comprehensive and systematic literature survey focusing on the integration of artificial intelligence (AI), machine learning (ML), and data-driven methodologies in the design and optimization of antimicrobial nanomaterials for food and biomedical applications. Relevant peer-reviewed articles were collected from major scientific databases, including Web of Science, Scopus, PubMed, ScienceDirect, and Google Scholar, covering publications, primarily from 2015 to 2026, to capture recent advances in computational materials science and antimicrobial nanotechnology.

The literature search was performed using combinations of keywords and Boolean operators, including “*artificial intelligence*”, “*machine learning*”, “*data-driven design*”, “*nanomaterials*”, “*antimicrobial nanoparticles*”, “*metal–organic frameworks*”, “*nanoemulsions*”, “*food safety*”, “*biomedical applications*”, “*materials informatics*”, and “*predictive modeling*”. Additional searches incorporated application-specific terms such as “*biofilm inhibition*”, “*nanotoxicity prediction*”, “*high-throughput screening*”, and “*autonomous materials discovery*” to ensure broad coverage of emerging interdisciplinary research areas.

Studies were included based on several criteria: (i) investigation of antimicrobial nanomaterials or nanostructured systems; (ii) application of AI-, ML-, or data-driven methodologies for prediction, optimization, classification, or mechanistic analysis; and (iii) relevance to food preservation, antimicrobial coatings, infection control, drug delivery, or related biomedical systems. Both experimental and computational studies were considered to provide balanced coverage of current developments. Review articles with significant conceptual contributions were also included to establish broader scientific context and identify major research trends. Studies were excluded if they: (i) focused exclusively on non-antimicrobial nanomaterials or conventional antimicrobial agents without a nanoscale component; (ii) did not incorporate artificial intelligence, machine learning, materials informatics, or other data-driven methodologies; (iii) lacked sufficient methodological details or relevant physicochemical and biological data; (iv) were conference abstracts, editorials, patents, book chapters, non-peer-reviewed publications, or articles not published in English; or (v) addressed applications unrelated to food safety or biomedical systems. Duplicate records identified across databases were removed prior to screening. The remaining studies were evaluated based on title, abstract, and full-text review to ensure relevance to the scope and objectives of this work. The literature selection process involved initial database retrieval, duplicate removal, title and abstract screening, full-text eligibility assessment, and final inclusion based on predefined criteria.

Following literature collection, studies were categorized according to nanomaterial class, computational methodology, and application domain. Nanomaterial systems were broadly classified into metal and metal oxide nanoparticles, polymeric nanomaterials, nanoemulsions, carbon-based nanomaterials, and metal–organic frameworks. Computational approaches were grouped into supervised learning, unsupervised learning, deep learning, generative models, and autonomous optimization systems. Particular emphasis was placed on studies that established relationships between physicochemical descriptors and antimicrobial performance, as these contributions are central to rational nanomaterial design.

In addition to summarizing current advances, this study critically evaluates the limitations associated with existing datasets, model interpretability, reproducibility, and scalability. Emerging trends involving explainable AI, multimodal data integration, and closed-loop experimental platforms are also highlighted to provide perspectives for future research directions in AI-enabled antimicrobial nanotechnology.

## 3. Artificial Intelligence and Data-Driven Approaches in Nanomaterials Research

### 3.1. Emergence of Artificial Intelligence in Materials Science

Artificial intelligence (AI) has rapidly emerged as a transformative technological framework capable of reshaping modern materials science and nanotechnology through accelerated discovery, predictive modeling, and autonomous optimization [15]. Historically, materials development has relied heavily on empirical experimentation, iterative synthesis, and trial-and-error methodologies, which often require substantial time, labor, and financial investment. Such limitations are particularly pronounced in nanomaterials research because nanoscale systems exhibit highly complex and nonlinear relationships between physicochemical characteristics and functional performance [16]. Minor variations in nanoparticle morphology, crystallinity, particle size distribution, surface chemistry, defect density, and elemental composition may significantly influence biological activity, catalytic efficiency, colloidal stability, and toxicological behavior. Consequently, conventional experimental optimization frequently becomes inefficient when addressing multidimensional nanoscale parameter spaces [17].

**Figure 1:**
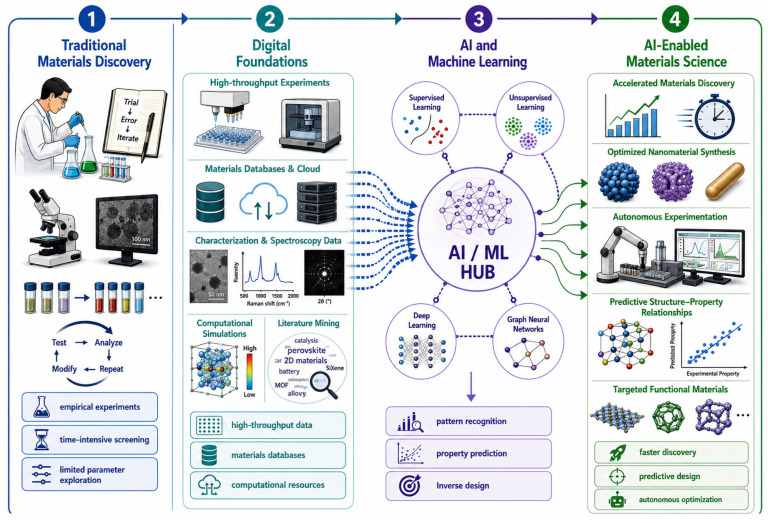
Emergence of artificial intelligence in materials science and nanotechnology.

The integration of AI and machine learning (ML) into materials science has provided a data-driven alternative that can identify hidden patterns and predictive relationships within large experimental and computational datasets [18]. Figure 1 illustrates the transition from conventional trial-and-error materials discovery toward AI-enabled materials science, in which high-throughput data, computational resources, materials databases, and machine learning models collectively support accelerated prediction, optimization, and autonomous discovery of functional nanomaterials.

Butler et al. (2018) described AI-assisted materials science as a paradigm shift in which algorithms can extract structure–property relationships that are difficult to identify using traditional statistical approaches [19]. Similarly, the expanding role of ML algorithms in accelerating materials discovery, property prediction, and inverse materials design has been highlighted [20]. In recent years, advances in computational infrastructure, high-throughput experimentation, cloud computing, and materials informatics have substantially increased the accessibility and applicability of AI-based methodologies across nanotechnology research [15].

Machine learning algorithms employed in nanomaterials research are generally classified into supervised learning, unsupervised learning, reinforcement learning, and deep learning frameworks. Supervised learning models utilize labeled datasets to predict targeted outputs such as antimicrobial activity, toxicity, adsorption capacity, or synthesis yield [20]. Common supervised learning algorithms include support vector machines (SVM), random forest (RF), decision trees, k-nearest neighbors (KNN), gradient boosting, and artificial neural networks (ANNs). These methods are widely used for quantitative prediction and classification tasks involving nanomaterial properties [21].

Unsupervised learning approaches are increasingly used for clustering analysis, dimensionality reduction, and identification of latent relationships within high-dimensional materials datasets [22]. Principal component analysis (PCA), hierarchical clustering, and t-distributed stochastic neighbor embedding (t-SNE) are among the most common approaches employed in nanotechnology [23]. These methods enable researchers to classify nanomaterials according to structural or functional similarities while improving the interpretation of complex datasets [24].

Deep learning represents one of the most rapidly advancing areas in AI-driven materials science. Convolutional neural networks (CNNs), recurrent neural networks (RNNs), and graph neural networks (GNNs) have demonstrated exceptional capability in image analysis, spectroscopy interpretation, and atomistic modeling [25]. CNN-based frameworks are increasingly integrated with electron microscopy and imaging systems to automate nanoparticle morphology characterization and defect identification [26]. GNNs are particularly valuable for modeling nanomaterials because they directly represent atomic interactions and bonding networks within graph-based architectures [27].

The rapid growth of AI-assisted nanotechnology has also been supported by the expansion of open-access materials databases and computational repositories. Large-scale initiatives, including the Materials Project, Open Quantum Materials Database (OQMD), AFLOWLIB, and NOMAD, have enabled access to extensive datasets containing structural, electronic, thermodynamic, and functional properties of diverse materials systems [28,29]. Such resources facilitate high-throughput computational screening and enable ML models to learn predictive relationships across broad chemical spaces.

In antimicrobial nanotechnology, AI methodologies are increasingly employed to predict antibacterial activity, optimize nanoparticle synthesis parameters, estimate nanotoxicity, and improve mechanistic understanding of nano–bio interactions [30]. Antimicrobial performance depends on numerous interconnected variables, including particle size, surface charge, ion release kinetics, reactive oxygen species (ROS) generation, hydrophobicity, and interaction with microbial membranes [31]. Machine learning algorithms provide an efficient strategy for analyzing these multidimensional relationships while reducing experimental burden [32].

Recent advances have highlighted the growing potential of artificial intelligence (AI)-assisted frameworks to enhance the prediction of nanoparticle performance and biological interactions [33]. For instance, David A. Winkler and co-workers demonstrated that machine learning-driven analysis of nanomaterial descriptors substantially improved the prediction of cellular uptake and nanotoxicity profiles. Similarly, emerging deep learning approaches have shown strong capability in predicting the antibacterial activity of nanoparticles by integrating physicochemical parameters with imaging-derived datasets [34,35]. Collectively, these developments underscore the increasing role of AI in accelerating the rational design, screening, and optimization of functional nanomaterials for biomedical and antimicrobial applications.

Despite these advances, several critical limitations continue to restrict the widespread implementation of AI-driven nanotechnology [34]. One major challenge involves the lack of standardized and high-quality datasets suitable for robust ML training [36]. Variability in synthesis protocols, characterization methods, biological assays, and reporting standards frequently results in inconsistent datasets with poor interoperability [37]. In addition, many AI models operate as black-box systems with limited mechanistic interpretability, reducing confidence in predictive outcomes and limiting translational application in industrial and regulatory settings [38].

Another emerging concern involves reproducibility and external validation. Many published ML models exhibit strong performance on internal datasets but demonstrate poor generalizability when applied to external experimental systems [20]. Such issues are especially problematic in biological applications where microbial strain variability, environmental conditions, and biofilm heterogeneity substantially influence antimicrobial outcomes [39]. To address these limitations, recent research increasingly emphasizes explainable AI (XAI), federated learning, multimodal data integration, and autonomous experimentation frameworks [40]. Explainable AI approaches aim to improve transparency and mechanistic interpretability by identifying the critical descriptors that drive predictive outcomes. Such strategies are expected to enhance reliability and accelerate translation of AI-guided nanomaterial design into practical food and biomedical applications [41,42].

### 3.2. Materials Informatics and Predictive Modeling of Nanomaterials

Materials informatics has emerged as one of the most influential interdisciplinary fields connecting materials science, computational modeling, data analytics, and artificial intelligence [43]. The primary objective of materials informatics is to accelerate materials discovery and optimization through data-driven analysis of structure–property–performance relationships [19]. In nanotechnology, materials informatics is particularly valuable because nanoscale systems involve highly multidimensional interactions among composition, morphology, surface chemistry, crystallinity, porosity, and environmental conditions [44].

Conventional optimization of nanomaterials often relies on extensive experimental trial-and-error approaches involving systematic adjustment of synthesis and post-processing conditions [45]. While effective to some extent, these strategies are time-consuming, labor-intensive, and frequently insufficient for comprehensively exploring the vast combinatorial design space associated with nanomaterial systems [46]. In response to these limitations, materials informatics has emerged as a powerful framework that integrates statistical learning and computational modeling to support predictive screening and rational material design [47]. Central to this approach is descriptor engineering, in which materials are represented through quantitative descriptors reflecting their structural, electronic, physicochemical, and functional properties [48]. In antimicrobial nanotechnology, widely utilized descriptors include particle size, morphology, surface area, zeta potential, hydrophobicity, crystallinity, oxidation state, ion release behavior, and band gap energy. The selection of relevant and informative descriptors is particularly critical, as the predictive performance and interpretability of machine learning models are strongly influenced by the quality and mechanistic relevance of the input variables [49,50].

A landmark contribution to quantitative nanostructure–activity relationship (QNAR) modeling was reported by Jerzy Leszczynski, Tomasz Puzyn, and colleagues in 2011, who demonstrated that electronic structure descriptors could effectively predict the cytotoxicity of metal oxide nanoparticles [51]. Their study provided one of the earliest robust frameworks for AI-assisted nanotoxicity assessment and demonstrated the potential of linking physicochemical characteristics with biological responses through computational modeling. Building upon this foundation, machine learning (ML)-assisted predictive approaches have increasingly been employed to estimate antimicrobial activity, nanoparticle stability, cellular internalization, and environmental toxicity [36]. These models are typically developed using supervised learning strategies trained on experimental datasets that integrate physicochemical descriptors with corresponding biological outcomes [52]. Among the available algorithms, random forest and gradient boosting models have gained substantial attention due to their strong predictive capability, robustness toward complex nonlinear relationships, and relatively high interpretability compared with many other AI-based approaches [53].

Recent advances in deep learning have substantially expanded the predictive and design capabilities of nanomaterials research. In particular, graph neural networks (GNNs) have emerged as powerful tools for modeling atomic-scale interactions by learning directly from structural representations of materials. These approaches are especially valuable for complex nanoscale systems, including metal–organic frameworks (MOFs), hybrid nanocomposites, and doped nanostructures, where conventional descriptor-based methods may struggle to fully capture structural complexity [54,55]. Notably, Tian Xie and Jeffrey C. Grossman demonstrated that crystal graph convolutional neural networks could accurately predict material properties directly from crystal structures without extensive manual feature engineering, highlighting the growing potential of data-driven representation learning in materials science [56]. Beyond predictive modeling, generative AI approaches have also attracted increasing attention for inverse nanomaterial design [57]. Frameworks such as variational autoencoders (VAEs), generative adversarial networks (GANs), and diffusion-based models enable the computational generation of hypothetical materials with targeted structural and functional characteristics [58]. These emerging strategies are now being actively explored for the development of advanced catalytic systems, energy-storage materials, and antimicrobial nanostructures with enhanced and optimized performance profiles [59].

Materials informatics has further advanced through its close integration with computational chemistry and atomistic simulation techniques, where approaches such as density functional theory (DFT), molecular dynamics (MD), and molecular docking collaboratively provide mechanistic insights and high-quality datasets that strengthen the predictive capability of machine learning models [60,61]. In particular, DFT-derived electronic descriptors have been widely incorporated into quantitative nanostructure–activity relationship (QNAR) frameworks to improve the prediction of oxidative stress generation, nanoparticle reactivity, and nanotoxicological behavior [51]. Simultaneously, the convergence of AI-driven analytics with computational modeling has substantially accelerated nanomaterial synthesis optimization, as Bayesian optimization strategies, Graph Neural Networks (GNNs) and evolutionary algorithms work synergistically to adaptively navigate complex experimental parameter spaces by iteratively identifying synthesis conditions most likely to maximize targeted performance outcomes [62,63]. Through this collaborative integration of computational prediction and experimental design, materials informatics not only reduces the number of required experimental iterations but also enhances optimization efficiency, reproducibility, and the overall rational development of advanced nanomaterial systems [64].

Another major advancement in materials informatics involves high-throughput computational screening supported by open-access databases such as Materials Project, AFLOWLIB, and Open Quantum Materials Database, which collectively provide extensive thermodynamic and electronic datasets for large-scale materials discovery [28,29]. Anubhav Jain and colleagues demonstrated that the Materials Project significantly accelerated computational screening of hypothetical compounds, and similar strategies are now increasingly applied to antimicrobial nanomaterials and MOF-based systems [65]. Despite these advances, predictive nanotechnology modeling remains limited by insufficient dataset standardization and incomplete metadata, as many studies lack detailed synthesis and characterization information required for robust machine learning training [66]. In addition, biological datasets often exhibit substantial variability due to differences in microbial strains, experimental conditions, exposure times, and assay protocols, which can reduce model reliability and generalizability [67].

Data imbalance remains a persistent challenge in predictive nanomaterial modeling, largely because negative or unsuccessful experimental outcomes are underreported in the scientific literature [68]. This publication bias can reduce model robustness, limit generalizability, and potentially lead to overestimated predictive performance. Consequently, the adoption of FAIR (Findable, Accessible, Interoperable, and Reusable) data principles has become increasingly important for improving dataset quality, transparency, and reproducibility in materials informatics [69]. Looking ahead, future advances in predictive nanotechnology are expected to increasingly rely on multimodal AI frameworks that integrate imaging, spectroscopy, genomics, and physicochemical datasets into unified analytical platforms. Such integrative approaches may substantially enhance the mechanistic understanding of antimicrobial nanomaterials while accelerating the rational development of highly optimized systems for targeted food safety and biomedical applications [70,71].

### 3.3. Autonomous Laboratories and Ai-Guided Experimental Platforms

The emergence of autonomous laboratories is among the most transformative advances in modern materials science and nanotechnology. These self-driving platforms integrate artificial intelligence, robotics, automated instrumentation, and real-time data analytics to establish closed-loop experimental workflows that can design, execute, evaluate, and optimize experiments with minimal human intervention [72]. By linking computational decision-making with automated experimentation, autonomous laboratories can substantially accelerate materials discovery while improving efficiency, reproducibility, and experimental consistency [73]. Nanomaterials research is particularly well-suited to this approach because nanoscale systems often involve highly complex, multidimensional design spaces that are difficult to explore using conventional trial-and-error methods [8]. In antimicrobial nanomaterial development, for instance, performance may depend on the simultaneous optimization of precursor composition, reaction temperature, pH, solvent ratio, surface functionalization, and post-synthesis processing [74,75]. Manually evaluating these interacting variables is not only time- and resource-intensive but also often insufficient for identifying globally optimal synthesis conditions.

AI-guided experimental platforms address the limitations of conventional nanomaterial optimization by using iterative, closed-loop learning workflows that connect computational prediction, automated synthesis, analytical characterization, and model refinement [76]. In a typical autonomous system, machine learning algorithms first analyze existing datasets to identify promising synthesis conditions, after which robotic platforms fabricate nanomaterials under the selected parameters and characterize the resulting products through spectroscopy, microscopy, diffraction, or biological assays [77]. The newly generated experimental data are then incorporated back into the model to guide the next experimental cycle, allowing the system to improve its predictions progressively [78]. A milestone was reported by Burger et al. (2020), who developed a mobile robotic chemist capable of independently conducting chemical synthesis and optimization experiments with minimal human supervision [79]. Their use of Bayesian optimization illustrated the strength of adaptive decision-making, in which the model balances exploration of uncertain regions with exploitation of conditions predicted to improve performance. This strategy is particularly valuable in nanomaterials research because it can reduce experimental burden while accelerating convergence toward optimized synthesis parameters [80].

Autonomous experimentation is further strengthened by the integration of high-throughput characterization with AI-assisted analytical tools. Convolutional neural networks, computer vision algorithms, and other deep learning frameworks are increasingly used to interpret SEM, TEM, AFM, XRD, and spectroscopic datasets with greater speed, consistency, and reproducibility than manual analysis alone [81,82]. This capability is especially important in antimicrobial nanotechnology, where particle morphology, aggregation behavior, crystallinity, defect density, and surface structure can strongly influence antimicrobial activity and biofilm inhibition [83]. By automatically extracting these features from imaging and characterization datasets, AI-assisted analysis reduces subjective bias, improves data standardization, and generates more reliable inputs for subsequent machine learning models [84]. In parallel, the integration of autonomous platforms with digital twins and cloud-based infrastructure is creating new opportunities for predictive experimentation [85]. Digital twins can serve as computational replicas of physical synthesis and characterization systems, enabling researchers to simulate experimental outcomes before conducting physical trials, thereby reducing material waste, improving resource efficiency, and supporting more rational experimental planning [86].

In antimicrobial nanotechnology, autonomous laboratories are increasingly relevant for optimizing nanoparticle synthesis, antibiofilm coatings, smart delivery systems, and photocatalytic antimicrobial materials for applications in food packaging, biomedical surfaces, and infection control [87]. These platforms also offer important advantages for reproducibility, as automated workflows can minimize variability associated with operator handling, inconsistent protocols, instrumentation differences, and environmental fluctuations [88]. Nevertheless, several challenges remain before autonomous laboratories can be broadly adopted, including high infrastructure costs, complex integration of robotics and analytical instruments, substantial computational requirements, and limited interoperability across hardware and software platforms [89,90]. Data governance, cybersecurity, algorithm transparency, and ownership of large experimental datasets also require careful consideration as cloud-connected laboratory systems become more common [91]. Future progress is expected to rely on the combined use of explainable AI, generative modeling, multimodal sensing, and edge computing, which may ultimately support fully autonomous discovery platforms capable of rapidly identifying antimicrobial nanomaterials tailored to foodborne pathogens, multidrug-resistant bacteria, and complex biofilm-associated infections [92,93].

## 4. Antimicrobial Nanomaterials and Their Functional Mechanisms

### 4.1. Metal and Metal Oxide Nanoparticles

Metal and metal oxide nanoparticles represent one of the most extensively investigated classes of antimicrobial nanomaterials because of their broad-spectrum activity, structural tunability, and ability to interact with microbial systems through multiple simultaneous mechanisms [31]. Among these materials, silver nanoparticles (AgNPs), zinc oxide nanoparticles (ZnO NPs), copper oxide nanoparticles (CuO NPs), titanium dioxide nanoparticles (TiO_2_ NPs), magnesium oxide nanoparticles, and iron oxide-based nanostructures have received substantial attention for food preservation, biomedical coatings, wound healing, water disinfection, and surface decontamination [94,95]. Their antimicrobial performance is generally attributed to a combination of physicochemical and biochemical interactions, including membrane disruption, reactive oxygen species (ROS) generation, ion release, protein oxidation, DNA damage, and interference with intracellular metabolic pathways. Recent reviews emphasize that metal and metal oxide nanoparticles remain among the most promising antimicrobial nanosystems, but their activity is strongly dependent on nanoparticle composition, surface chemistry, particle size, microbial strain, exposure conditions, and biofilm state [96].

Silver nanoparticles are frequently considered benchmark antimicrobial nanomaterials because of their strong activity against Gram-positive bacteria, Gram-negative bacteria, fungi, and some viruses [97]. Their antimicrobial action is commonly associated with the release of Ag^+^ ions, which can bind to thiol groups in proteins, disrupt respiratory enzymes, interfere with membrane permeability, and damage nucleic acids. In addition, AgNPs can interact directly with bacterial membranes, increasing membrane permeability and causing leakage of intracellular components [98,99]. The high surface-area-to-volume ratio of nanoscale silver enhances interfacial contact with microbial cells, thereby improving antimicrobial efficiency compared with bulk silver or conventional silver salts. However, AgNP activity is not universal; it varies substantially with particle size, surface coating, aggregation state, and medium composition [75]. Organic matter, proteins, and salts in food or biological systems may reduce free Ag^+^ availability and alter antimicrobial performance [100].

ZnO nanoparticles have also attracted considerable interest because of their relatively low cost, broad-spectrum antibacterial activity, photocatalytic potential, and relevance to food packaging and biomedical surfaces. ZnO NPs inhibit microorganisms through ROS generation, Zn^2+^ release, membrane interaction, and oxidative damage. The antibacterial activity of ZnO is strongly influenced by particle size, morphology, crystallinity, defect density, and light exposure. Oxygen vacancies and surface defects may enhance ROS generation, while smaller particles generally provide greater surface reactivity [101]. Recent reviews have described ZnO NPs as promising inorganic antimicrobial agents because they combine chemical stability with multiple bactericidal pathways [102,103].

TiO_2_ nanoparticles have attracted considerable attention for photocatalytic antimicrobial applications because they can generate highly reactive oxygen species, including hydroxyl radicals and superoxide anions, under ultraviolet or visible-light activation. These reactive species can oxidatively damage microbial membranes, proteins, and nucleic acids, thereby supporting the development of self-cleaning surfaces, antimicrobial coatings, and water-disinfection technologies. Despite these advantages, the dependence of TiO_2_ on light activation may restrict its effectiveness under low-light or dark environments, prompting growing interest in doped or modified TiO_2_ systems that can extend photocatalytic activity into the visible-light region [104].

Copper oxide and related metal oxide nanoparticles provide additional antimicrobial mechanisms through Cu^2+^ release, oxidative stress induction, and membrane disruption [105]. Copper-based nanomaterials are particularly attractive because they are more cost-effective than silver while still exhibiting broad-spectrum antimicrobial activity. However, uncontrolled copper ion release may introduce cytotoxicity and environmental accumulation concerns, underscoring the need for careful dose optimization, controlled-release design, and surface engineering to balance antimicrobial efficacy with biological and ecological safety.

Despite their promise, metal and metal oxide nanoparticles present several translational challenges. Strong antimicrobial activity is often associated with high surface reactivity, but the same property may contribute to mammalian cytotoxicity, oxidative stress, or ecological risk [106]. Therefore, modern design strategies increasingly focus on safe-by-design approaches, including polymer coating, dopant control, controlled-release architectures, hybridization with biopolymers, and AI-guided optimization [107]. Data-driven modeling can help identify material compositions and physicochemical windows that maximize antimicrobial efficacy while limiting toxicity. This balance is essential for applications in food-contact materials, wound dressings, medical implants, and environmental disinfection systems.

### 4.2. Metal–Organic Frameworks and Hybrid Nanostructures

Metal–organic frameworks (MOFs) have emerged as highly versatile antimicrobial nanomaterials because of their crystalline porous structures, tunable chemistry, high surface area, and capacity to host or release antimicrobial agents [108]. MOFs are composed of metal ions or clusters coordinated with organic linkers, producing framework architectures that can be engineered for controlled release, surface functionality, adsorption, catalysis, and biological interaction. In antimicrobial research, MOFs are attractive because their activity can arise from both intrinsic framework components and externally loaded antimicrobial compounds [109]. Recent reviews describe MOFs and MOF-based materials as promising broad-spectrum antimicrobial systems with potential activity against bacteria, fungi, viruses, and biofilms [110].

Among MOFs, zeolitic imidazolate framework-8 (ZIF-8) is one of the most widely studied for antimicrobial applications [111]. ZIF-8 consists of Zn^2+^ nodes coordinated with 2-methylimidazolate linkers and is valued for its relatively high stability, pH-responsive degradation, and capacity to encapsulate bioactive molecules. Its antimicrobial activity is commonly attributed to Zn^2+^ release, membrane interaction, intracellular stress, and controlled delivery of antimicrobial guests. ZIF-8 can also act as a carrier for antibiotics, essential oils, enzymes, photosensitizers, or metal nanoparticles, allowing the construction of multifunctional antimicrobial platforms [112,113]. The recent literature highlights ZIF-8 and its derivatives as particularly important antibacterial MOF systems because their structure can be modified with metals, polymers, antibiotics, and other functional components to enhance activity against Gram-positive and Gram-negative bacteria [114,115].

Hybrid MOF-based nanostructures are especially important because they combine multiple antimicrobial mechanisms within a single material [116]. For example, Ag-loaded MOFs may integrate Ag^+^-mediated bacterial inactivation with MOF-based controlled release. MOF–polymer composites can improve colloidal stability, reduce premature degradation, and enhance adhesion to biological or food-contact surfaces [117,118]. Essential-oil-loaded MOFs can protect volatile hydrophobic compounds from rapid evaporation or oxidation while enabling sustained antimicrobial release [119]. Similarly, MOF–photothermal or MOF–photodynamic systems may combine chemical and light-triggered antimicrobial activity, which is highly relevant for wound healing, implant disinfection, and biofilm treatment [120].

The antimicrobial mechanisms of MOFs can be classified into several major pathways [116]. First, metal ion release from MOFs can disrupt bacterial membranes, interfere with metabolic enzymes, and induce oxidative stress. Second, the porous framework may serve as a nanocarrier for sustained release of antimicrobial compounds [108]. Third, MOFs with catalytic or photocatalytic properties may generate ROS under appropriate stimulation [121]. Fourth, surface-modified MOFs can interact with bacterial membranes or biofilm matrices, enhancing local concentration and contact-mediated killing [122]. Finally, MOFs can be engineered for stimuli-responsive behavior, including pH-, light-, heat-, or redox-triggered release [123]. This is particularly useful because infection sites, biofilms, and food spoilage microenvironments often present altered pH, enzyme activity, and oxidative conditions.

However, MOF-based antimicrobial systems also face important limitations. Many MOFs exhibit variable stability in aqueous, acidic, saline, or protein-rich environments [124]. Framework degradation may be beneficial for controlled release but problematic if it causes uncontrolled ion release or loss of structure before reaching the target site. Potential toxicity from metal ions, organic linkers, or degradation products must be carefully evaluated [125]. In addition, large-scale production, batch reproducibility, cost, and regulatory acceptance remain major barriers for food and biomedical translation [126]. These challenges make MOFs highly suitable candidates for AI-assisted design, because their large compositional and structural diversity cannot be efficiently optimized by trial-and-error methods alone [55]. Machine learning can support screening of metal nodes, linkers, pore structures, guest molecules, and surface modifications to identify MOF systems with improved antimicrobial performance, stability, and safety [127].

### 4.3. Polymeric Nanocarriers, Nanoemulsions, and Soft Nanomaterials

Polymeric nanocarriers, nanoemulsions, liposomes, nanogels, dendrimers, and related soft nanomaterials represent another important class of antimicrobial nanosystems. Unlike many inorganic nanoparticles, these platforms are often designed to encapsulate, stabilize, and deliver antimicrobial compounds rather than relying solely on the intrinsic toxicity of the material [128]. Their advantages include tunable release kinetics, improved solubility of hydrophobic compounds, reduced volatility, enhanced bioavailability, and compatibility with food and biomedical environments [129]. Recent studies have emphasized the growing role of polymeric nanoparticles and nanoparticle–hydrogel systems in antimicrobial delivery, wound healing, infection control, and antibiotic-resistance management [130]. Table 1 summarizes the major polymeric nanocarrier and nanoemulsion-based antimicrobial systems, highlighting their functional mechanisms, application potential, and key limitations in food and biomedical contexts.

Chitosan-based nanoparticles are among the most important polymeric antimicrobial nanomaterials. Chitosan is a cationic, biodegradable polysaccharide that can interact electrostatically with negatively charged bacterial membranes. This interaction may increase membrane permeability, disrupt ion transport, and promote leakage of intracellular components [131]. Chitosan can also serve as a carrier for essential oils, antibiotics, phenolic compounds, enzymes, and metal nanoparticles. Its functional groups enable chemical modification and crosslinking, thereby allowing control over particle size, surface charge, mucoadhesion, and release behavior [141]. In food applications, chitosan nanoparticles are frequently investigated for edible coatings, antimicrobial films, and produce preservation. In biomedical systems, they are explored for wound dressings, drug delivery, and infection control [142].

Nanoemulsions are particularly useful for antimicrobial compounds with poor aqueous solubility, including essential oils and hydrophobic plant-derived antimicrobials. These systems consist of nanoscale oil droplets dispersed in an aqueous phase and stabilized by surfactants or biopolymers [143]. Essential oil nanoemulsions containing compounds such as thymol, carvacrol, eugenol, cinnamaldehyde, tea tree oil, or oregano oil can enhance antimicrobial activity by improving dispersion, increasing contact area, and facilitating interaction with microbial membranes [144]. Their nanoscale droplet size may also improve penetration into biofilms or food surface microstructures. However, nanoemulsion performance depends strongly on droplet size, polydispersity, interfacial composition, surfactant type, storage stability, pH, ionic strength, and matrix interactions [145].

Hydrogels incorporating antimicrobial nanoparticles have also become important in biomedical applications. These hybrid systems combine the moisture-retaining and tissue-compatible properties of hydrogels with the antimicrobial activity of nanoparticles or loaded agents [146]. They are particularly relevant for wound healing because they can provide sustained antimicrobial release, maintain a moist wound environment, absorb exudate, and reduce bacterial colonization [147]. Nanoparticle–hydrogel systems may incorporate silver nanoparticles, ZnO nanoparticles, antibiotics, antimicrobial peptides, or natural compounds [148]. Recent reviews describe these systems as promising platforms for controlled, sustained, and targeted antimicrobial delivery.

Soft nanomaterials are also attractive for biofilm control because they can be engineered for penetration, adhesion, and localized release [149]. Biofilms present major barriers to antimicrobial treatment due to extracellular polymeric substances, altered microbial metabolism, and diffusion limitations [150]. Polymeric nanoparticles, liposomes, dendrimers, and nanogels can be functionalized to improve biofilm penetration or respond to biofilm-associated triggers such as acidic pH, enzymes, or oxidative stress [151]. Recent reviews on nanomaterial-enabled antibiofilm strategies highlight nano-delivery systems, including liposomes, nanoemulsions, polymers, dendrimers, nanogels, inorganic nanoparticles, and MOFs, as promising tools for overcoming biofilm resistance [152].

Despite these advantages, polymeric and soft nanomaterials still face challenges in formulation and translation. Their stability may be affected by temperature, pH, ionic strength, proteins, enzymes, and storage conditions. In food systems, sensory impact, ingredient compatibility, migration behavior, and regulatory approval are major concerns [153]. In biomedical systems, degradation behavior, immune response, sterility, and reproducibility must be carefully controlled [154,155]. Data-driven optimization is therefore particularly valuable for these systems, because formulation variables interact nonlinearly and influence multiple outcomes simultaneously, including size, encapsulation efficiency, release kinetics, antimicrobial activity, and cytotoxicity [156].

### 4.4. Antibiofilm Mechanisms and Nano–Bio Interactions

Biofilms are structured microbial communities embedded in extracellular polymeric substances that protect microorganisms from antimicrobial agents, immune responses, and environmental stress [157]. Biofilm-associated contamination and infection are major concerns in food-processing environments, water systems, medical devices, chronic wounds, and implant-associated infections [158]. Compared with planktonic cells, biofilm microorganisms exhibit increased tolerance due to diffusion barriers, altered metabolism, persister cell formation, quorum sensing, and protective extracellular matrices. For this reason, antibiofilm activity is an essential performance criterion for antimicrobial nanomaterials [159]. Recent reviews emphasize that nanomaterials offer unique advantages for biofilm control because their small size, surface tunability, and multifunctional mechanisms can improve penetration, adhesion, and localized antimicrobial action [160].

**Figure 2:**
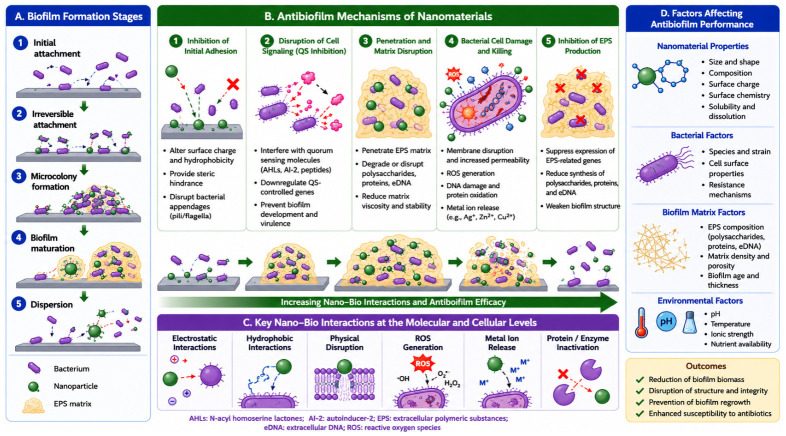
Antibiofilm mechanisms and nano–bio interactions of antimicrobial nanomaterials.

Nanomaterials can inhibit biofilms at multiple stages of biofilm development. Figure 2 summarizes the major antibiofilm mechanisms of antimicrobial nanomaterials and highlights how nano–bio interactions influence microbial adhesion, biofilm disruption, extracellular polymeric substance degradation, and bacterial inactivation across different stages of biofilm development.

During the initial adhesion stage, antimicrobial coatings or nanoparticle-modified surfaces may reduce bacterial attachment by altering surface charge, hydrophobicity, or roughness, or by increasing contact-killing capacity [161]. During biofilm maturation, nanoparticles, such as PEG-coated ciprofloxacin-loaded ZIF-8, may penetrate the extracellular polymeric matrix, generate ROS, release antimicrobial ions or drugs, and disrupt cell–cell communication [162]. During established biofilm treatment, nanomaterials may weaken the matrix, kill embedded bacteria, or enhance susceptibility to conventional antimicrobials [163]. These multi-stage mechanisms distinguish nanomaterials from many conventional antimicrobials that rely primarily on diffusion and single-target biochemical inhibition.

Metal nanoparticles and metal oxides often act against biofilms through oxidative stress, ion release, and direct membrane damage [164]. AgNPs may penetrate biofilm matrices and release Ag^+^ ions locally, while ZnO and TiO_2_ nanoparticles may generate ROS that oxidize extracellular polymers and microbial membranes [75,102]. Copper-based nanoparticles may damage membranes and proteins through Cu^2+^ release and redox cycling [165]. However, the extracellular polymeric matrix can also bind nanoparticles, reduce ion availability, or prevent deep penetration, meaning antibiofilm performance is strongly dependent on particle size, surface charge, and coating chemistry [166].

Polymeric nanoparticles, liposomes, and nanoemulsions can serve as carriers for antibiofilm agents such as antibiotics, essential oils, enzymes, quorum-sensing inhibitors, or antimicrobial peptides [152]. These systems may improve penetration into biofilms and protect loaded agents from degradation. Stimuli-responsive nanocarriers are especially promising because they can release antimicrobial payloads in response to acidic pH, bacterial enzymes, redox conditions, or light [167]. MOFs and hybrid nanostructures similarly offer controlled release and multifunctional activity, combining ion release, guest molecule delivery, and catalytic ROS generation [116]. Nano–bio interactions are strongly influenced by both nanomaterial and microbial characteristics. Particle size affects penetration and surface area; surface charge influences interaction with bacterial membranes and extracellular polymeric substances; hydrophobicity affects adhesion; and surface coatings determine colloidal stability and biological identity [168]. Gram-positive and Gram-negative bacteria respond differently because of differences in cell wall architecture. Gram-negative bacteria possess an outer membrane containing lipopolysaccharides, whereas Gram-positive bacteria contain a thick peptidoglycan layer. Fungal cells and spores present additional structural barriers [169,170]. Therefore, antimicrobial nanomaterial design must consider organism-specific cell envelope structure and biofilm organization.

For food and biomedical systems, antibiofilm performance must be evaluated under realistic conditions. Food residues, proteins, fats, minerals, and surface roughness can substantially alter biofilm architecture and nanoparticle activity [171]. In biomedical environments, serum proteins, immune components, tissue fluids, and extracellular matrix molecules can modify nanoparticle behavior through protein corona formation or surface fouling [172]. Therefore, MIC values from planktonic cultures should not be used alone to predict practical antibiofilm performance [173]. Future studies should integrate biofilm-specific assays, microscopy, live/dead imaging, matrix quantification, and omics-based stress-response analysis to better understand nanomaterial effects on biofilm systems.

AI and data-driven tools can further strengthen antibiofilm nanomaterial design by linking nanomaterial descriptors to biofilm outcomes. Predictive models could incorporate particle size, zeta potential, release kinetics, matrix composition, biofilm thickness, bacterial species, and environmental conditions to identify design rules for biofilm penetration and disruption [174]. Such approaches are essential because biofilm systems are highly heterogeneous and difficult to optimize empirically. Ultimately, the most effective antimicrobial nanomaterials will be those that combine strong planktonic killing, antibiofilm activity, matrix compatibility, safety, and stability under realistic application conditions [160].

## 5. Data-Driven Design of Antimicrobial Nanomaterials

### 5.1. Descriptor-Based Prediction of Antimicrobial Activity

The data-driven design of antimicrobial nanomaterials relies on the ability to convert complex nanoscale properties into measurable, computable, and biologically meaningful descriptors. In machine learning (ML)-assisted nanomaterial design, descriptors function as input variables that allow algorithms to identify relationships between material structure, synthesis conditions, exposure parameters, and antimicrobial outcomes [36]. For antimicrobial nanomaterials, commonly used descriptors include particle size, morphology, aspect ratio, surface area, crystallinity, elemental composition, dopant concentration, oxidation state, surface charge, hydrophobicity, band gap energy, ion release behavior, coating chemistry, aggregation state, dose, exposure time, and microbial species [32]. These descriptors are important because antimicrobial activity is rarely controlled by a single variable. Instead, it often emerges from nonlinear interactions among surface reactivity, membrane affinity, reactive oxygen species (ROS) generation, metal ion release, dissolution kinetics, biofilm penetration, and organism-specific susceptibility [168].

Early data-driven approaches in nanotoxicology and antimicrobial nanotechnology were influenced by quantitative structure–activity relationship (QSAR) and quantitative nanostructure–activity relationship (QNAR) modeling [175]. These frameworks attempt to correlate physicochemical descriptors with biological outcomes such as cytotoxicity, oxidative stress, cellular uptake, bacterial inhibition, and organism-level toxicity. Puzyn et al. (2011) provided an important foundation for nano-QSAR modeling by demonstrating that metal oxide nanoparticle cytotoxicity could be predicted from electronic structure descriptors [51]. Although this study focused on cytotoxicity rather than antibacterial efficacy, it established a critical principle for nanomaterial informatics: nanoscale biological responses can be modeled from carefully selected physicochemical features [176].

**Figure 3:**
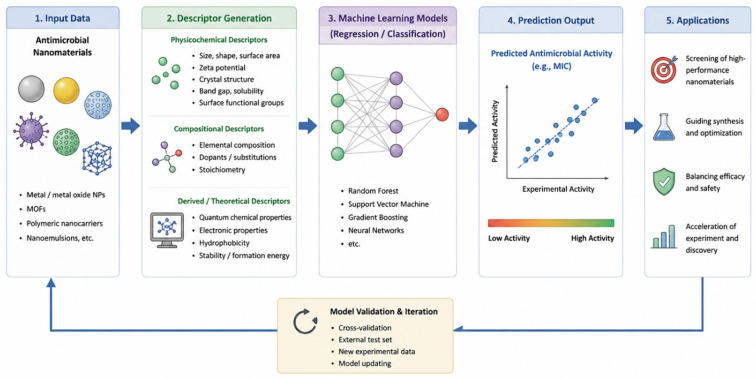
Descriptor-based prediction workflow for antimicrobial nanomaterials.

Figure 3 illustrates a descriptor-based machine learning workflow for predicting antimicrobial activity, where physicochemical, compositional, and theoretical descriptors of nanomaterials are integrated with predictive models to support rational antimicrobial nanomaterial design. More recently, descriptor-based ML has been applied directly to antimicrobial nanomaterials. Mirzaei et al. [32] developed a machine learning tool to predict the antibacterial activity of nanoparticles using data from 60 in vitro studies. Their model incorporated nanoparticle physicochemical properties, exposure conditions, and bacterial classification, and reported encouraging validation performance, with an R^2^ value of approximately 0.78. This study is particularly important because it showed that antibacterial prediction requires integrating both material descriptors and biological–experimental descriptors, rather than relying solely on nanoparticle properties. In other words, the same nanomaterial may show different levels of antimicrobial activity depending on bacterial strain, Gram classification, exposure duration, culture medium, assay method, and applied concentration [177].

Metal oxide nanoparticles have become especially important in descriptor-based antimicrobial modeling because their activity is strongly influenced by structural and electronic features, including crystallinity, oxygen vacancies, band gap energy, dopant identity, and surface defect density. Perfecto-Avalos et al. [178] employed statistical and ML models to investigate how cerium doping influences the antibacterial behavior of ZnO nanoparticles. Their study linked dopant incorporation with antibacterial performance, supporting the broader concept that ML can help identify composition–activity relationships in doped antimicrobial nanomaterials. Similarly, Navarro-López et al. [179] used ML approaches to predict bacterial survival against ZnO and lanthanum-doped ZnO nanoparticles, showing that data-driven models can help interpret how dopant chemistry affects antimicrobial response against different bacterial species.

A key advantage of descriptor-based modeling is its capacity to identify which material or experimental variables most strongly influence antimicrobial performance. Feature importance analysis from random forest, gradient boosting, or explainable AI methods can reveal whether bacterial inhibition is primarily associated with particle size, dopant concentration, surface charge, bacterial type, nanoparticle dose, or exposure time [30]. Such information is important because it allows ML models to move beyond simple prediction and toward mechanistic interpretation. For example, if particle size and zeta potential dominate prediction in polymeric nanoparticles, membrane adhesion and cellular contact may be the key antimicrobial determinants. In contrast, if dopant level, band gap energy, and crystallinity dominate prediction in metal oxides, ROS generation and defect-mediated oxidative stress may be more important [180].

Despite these advantages, descriptor-based prediction remains limited by heterogeneity in the antimicrobial nanomaterial literature. Studies frequently differ in nanoparticle synthesis method, purification protocol, characterization depth, bacterial strain, inoculum density, growth medium, exposure duration, temperature, endpoint measurement, and data reporting [181]. Some studies report inhibition zones, whereas others report minimum inhibitory concentration (MIC), minimum bactericidal concentration (MBC), optical density reduction, colony-forming unit reduction, biofilm biomass inhibition, or live and dead staining [114,182]. These endpoints are not always directly comparable. As a result, ML models trained on heterogeneous datasets may learn assay-specific patterns rather than generalizable antimicrobial design rules.

For food and biomedical applications, descriptor-based models must also include environmental and matrix-related variables. In food systems, proteins, fats, carbohydrates, salts, organic matter, and pH can alter nanoparticle aggregation, ion release, ROS generation, and microbial contact [183]. In biomedical systems, serum proteins, extracellular polymeric substances, tissue fluids, immune components, and protein corona formation can substantially change nanomaterial behavior [184]. Future descriptor frameworks should move beyond simplified nanoparticle-only datasets and incorporate three descriptor levels: material descriptors, biological descriptors, and application-environment descriptors. This integrated approach would allow a more realistic prediction of antimicrobial performance under practical food and biomedical conditions.

### 5.2. Machine Learning-Guided Synthesis and Formulation Optimization

In addition to predicting antimicrobial activity, data-driven strategies are increasingly used to optimize nanomaterial synthesis and formulation. Nanomaterial properties are highly sensitive to synthesis variables such as precursor concentration, solvent composition, reducing agent, stabilizer type, pH, reaction temperature, mixing speed, reaction time, ionic strength, and post-synthesis processing [185]. These variables determine particle size distribution, morphology, crystallinity, surface chemistry, aggregation behavior, porosity, and release kinetics. As antimicrobial performance is directly connected to these properties, synthesis optimization is a central component of antimicrobial nanomaterial design.

Traditional optimization often relies on one-factor-at-a-time experimentation or response surface methodology. In addition, these approaches may be insufficient for complex nanomaterial systems because they do not fully capture nonlinear interactions among synthesis variables. ML-guided optimization offers a more flexible approach by learning relationships between synthesis conditions and target outputs, such as optical response, antimicrobial activity, encapsulation efficiency, cytotoxicity, or stability [186]. This enables researchers to identify optimized synthesis conditions with fewer experiments than exhaustive screening.

**Figure 4:**
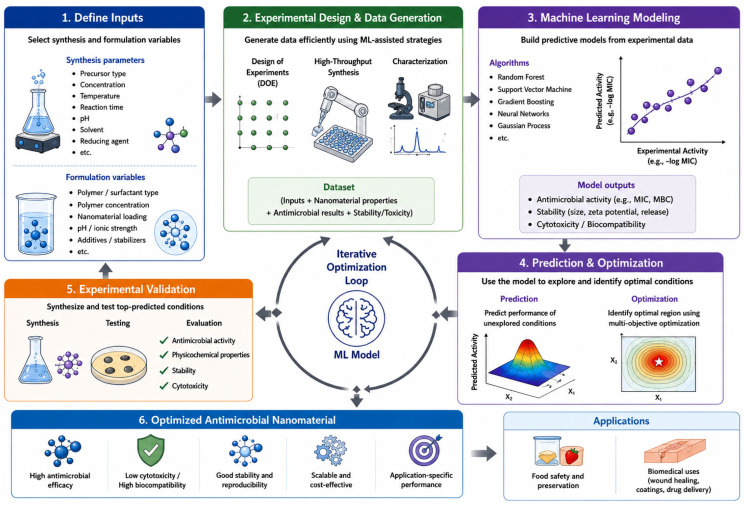
Closed-loop machine learning framework for the predictive design and optimization of antimicrobial nanomaterials.

Figure 4 presents a machine learning-guided workflow for antimicrobial nanomaterial synthesis and formulation optimization, integrating experimental design, predictive modeling, iterative validation, and multi-objective optimization to accelerate application-specific material development. Bayesian optimization has become particularly valuable in nanomaterial synthesis because it can efficiently explore experimental parameter spaces under limited-data conditions. Mekki-Berrada et al. [187] demonstrated a two-step ML framework for optimized nanoparticle synthesis using a high-throughput microfluidic platform. Their approach used Gaussian process-based Bayesian optimization and deep neural networks to produce silver nanoparticles with desired optical properties, while learning how chemical process variables influenced nanoparticle behavior. Although the targeted output in that study was optical absorbance rather than antibacterial activity, the framework is highly relevant to antimicrobial nanomaterials because size, morphology, surface chemistry, and optical/plasmonic properties are often linked to biological performance.

High-throughput and closed-loop experimental platforms further strengthen ML-guided synthesis. Park et al. [188] described a closed-loop nanoparticle synthesis pipeline integrating robotic synthesis, automated characterization, ML optimization, and computational prediction of structure–property relationships. Such systems demonstrate how nanoparticle synthesis can be transformed from manual trial-and-error work into an iterative self-optimizing workflow. In antimicrobial nanotechnology, this strategy could be extended to optimize metal nanoparticles, metal oxide nanoparticles, MOFs, polymeric nanocarriers, and nanoemulsions by linking synthesis variables to antimicrobial activity, antibiofilm efficacy, mammalian cell viability, and formulation stability [189].

ML-guided synthesis is especially useful when multiple performance objectives must be balanced. A practical antimicrobial nanomaterial should not only inhibit microbial growth but also remain stable, dispersible, reproducible, safe, scalable, and compatible with the intended application matrix. For example, increasing Ag^+^ release may enhance antimicrobial activity but may also increase mammalian cytotoxicity or migration concerns in food packaging [190]. Similarly, reducing particle size may increase bacterial contact but may also increase aggregation, oxidative reactivity, or biological uptake. Multi-objective optimization can help identify design windows where antimicrobial efficacy is maximized while toxicity, instability, and cost are minimized [191].

Machine learning is also highly relevant for polymeric nanocarriers and nanoemulsions. These systems are commonly designed to encapsulate hydrophobic antimicrobial compounds, essential oils, phenolics, antibiotics, antimicrobial peptides, or metal nanoparticles. Their performance depends on formulation variables such as polymer concentration, oil phase composition, surfactant type, homogenization speed, ionic strength, crosslinking density, and encapsulation conditions [192]. Output variables may include droplet size, polydispersity index, zeta potential, encapsulation efficiency, release profile, thermal stability, antimicrobial activity, and cytotoxicity. Because these variables interact nonlinearly, ML models can be more powerful than conventional statistical optimization.

In food applications, ML-guided formulation could support the development of antimicrobial nanoemulsions for edible coatings, fresh-produce washing, active packaging, and food-contact surface treatments [193]. The model could simultaneously optimize antimicrobial efficacy, sensory acceptability, compound retention, release behavior, and migration risk. In biomedical applications, similar approaches could be used to optimize wound dressings, implant coatings, drug-loaded nanoparticles, and antibiofilm delivery systems by balancing antimicrobial potency, sustained release, tissue compatibility, and inflammatory response.

A key challenge is that biological assays are slower and more variable than physical characterization methods. While UV–Vis spectroscopy, DLS, zeta potential, and microscopy can be automated relatively easily, antimicrobial assays often require microbial culture, incubation, plating, imaging, or biofilm quantification [194]. Advances in microfluidics, automated liquid handling, high-content imaging, and AI-assisted image analysis can help overcome this limitation. Ortiz-Perez et al. [195] demonstrated that active ML combined with high-throughput nanoparticle formulation and high-content imaging can accelerate nanoparticle design, supporting the broader feasibility of integrated AI-guided formulation pipelines. Table 2 summarizes the major machine learning-guided optimization strategies used to improve synthesis efficiency, formulation performance, and application-specific functionality of antimicrobial nanomaterials.

Compared with conventional trial-and-error approaches, AI-assisted design strategies offer significant advantages for the development of antimicrobial nanomaterials. Traditional experimental workflows typically rely on sequential optimization of individual synthesis parameters, requiring extensive experimental resources and often overlooking complex interactions among material properties. In contrast, machine learning models can simultaneously evaluate multiple descriptors, including particle size, surface charge, composition, dopant concentration, release kinetics, and biological response. For example, Bayesian optimization frameworks have been successfully applied to silver nanoparticle synthesis to identify optimal reaction conditions with substantially fewer experimental iterations than conventional approaches [196]. Machine learning models have been used to predict the antibacterial performance of doped ZnO nanoparticles by linking physicochemical descriptors with microbial inhibition outcomes, enabling more efficient screening of compositional variations. Graph-based learning approaches have also emerged as promising tools for screening metal–organic frameworks with targeted antimicrobial properties, reducing the need for exhaustive experimental synthesis [63]. These examples demonstrate that AI-assisted methodologies can accelerate materials discovery, improve reproducibility, and facilitate multi-objective optimization of antimicrobial efficacy, safety, and stability.

Overall, ML-guided synthesis and formulation optimization provide a rational pathway for developing antimicrobial nanomaterials with improved reproducibility, scalability, and application-specific performance. The most promising future workflows will combine automated synthesis, standardized characterization, antimicrobial testing, safe-by-design screening, and explainable ML into closed-loop platforms that continuously learn from experimental outcomes.

### 5.3. AI-Assisted Nanotoxicity Prediction and Safe-by-Design Development

The translation of antimicrobial nanomaterials into food and biomedical applications requires careful balancing of microbial inhibition with safety for human cells, food systems, and the environment. Nanomaterials that exhibit strong antimicrobial activity may also induce mammalian cytotoxicity, oxidative stress, inflammation, genotoxicity, immune activation, or ecological toxicity [199]. This is particularly important because many antimicrobial mechanisms, including ROS generation, membrane disruption, and metal ion release, are not exclusively selective toward microbial cells. Therefore, AI-assisted nanotoxicity prediction has become a critical component of safe-by-design antimicrobial nanomaterial development.

AI-assisted nanotoxicology uses ML models to predict toxicological outcomes based on physicochemical descriptors, exposure variables, biological context, and experimental endpoints. Early nano-QSAR and QNAR models showed that nanoparticle toxicity could be correlated with descriptors such as size, surface area, chemical composition, oxidation state, surface charge, and electronic properties [176]. Since then, more advanced ML methods, including random forest, support vector machines, gradient boosting, neural networks, and AutoML pipelines, have been applied to predict cytotoxicity, cellular uptake, oxidative stress, inflammatory response, and organism-level toxicity.

Recent studies highlight the growing role of AI in advancing nanotoxicology. Singh et al. [200] comprehensively demonstrated that AI and ML techniques in nanomedicine and nanotoxicology, emphasizing their potential to improve understanding of nanoscale toxic effects and support safer nanomaterial development. Similarly, Campagnolo et al. argued that AI can improve in vitro nanotoxicology by identifying patterns in large datasets that may be missed by traditional analyses [201]. Khokhlov et al. (2024) also noted that modern ML models can effectively classify nanoparticle toxicity categories and predict cell viability outcomes, while underscoring persistent challenges associated with limited dataset size, data heterogeneity, and variable reporting standards [202].

Recent advances have also introduced automated data extraction for nanotoxicity prediction. Ha et al. [203] developed an AI-based automated data extraction pipeline using large language model-assisted workflows to collect and organize nanotoxicity information from unstructured literature. Their study used nanotoxicity research articles as training material and developed prediction models for nanomaterial toxicity. This type of approach is important because nanotoxicity data are often dispersed across publications with inconsistent terminology, formatting, and metadata completeness. Automated extraction can help build larger and more standardized datasets for future predictive modeling.

For antimicrobial nanomaterials, safe-by-design development requires simultaneous optimization of efficacy and toxicity. Strong antimicrobial activity may be associated with high ROS production, rapid ion release, or strong membrane interaction, but these features may also damage mammalian cells or disrupt beneficial microbiota [204]. AI models can help identify design windows where antimicrobial activity is sufficient while cytotoxicity remains within acceptable limits. For example, a model may suggest that controlled Ag^+^ release or moderate Zn^2+^ dissolution provides bacterial inhibition without excessive mammalian toxicity. Similarly, polymer coatings, biopolymer encapsulation, or surface charge modulation may reduce nonspecific cytotoxicity while maintaining antimicrobial function [154].

Explainable AI tools are especially important in this context. If a predictive model identifies a nanomaterial as potentially toxic, interpretation methods such as SHAP, LIME, permutation importance, or partial dependence analysis can help determine whether toxicity is driven by dose, particle size, surface charge, dissolution rate, elemental composition, or exposure duration [205]. This information can guide redesign strategies, such as increasing coating stability, reducing dopant concentration, modifying surface charge, increasing particle size, or controlling release kinetics.

Food applications introduce additional safety requirements. Antimicrobial nanomaterials used in active packaging, edible coatings, or produce washing systems must be evaluated for migration, ingestion exposure, food matrix interactions, sensory effects, and potential impacts on beneficial microorganisms [206]. On the other hand, biomedical applications require evaluation of hemocompatibility, immunogenicity, tissue compatibility, degradation products, and long-term persistence [207]. Therefore, predictive toxicity models should not be generic; they should be tailored to exposure route, target application, and biological context.

Environmental safety is another important consideration. Antimicrobial nanomaterials may enter wastewater, soil, and aquatic ecosystems through manufacturing, disposal, washing, or biomedical waste streams [208]. Metallic and metal oxide nanoparticles can affect environmental microbial communities, algae, invertebrates, and biofilm-based ecosystems. AI-assisted ecological risk modeling can help predict organism-specific toxicity and environmental persistence, but this requires datasets that include environmental parameters such as pH, salinity, natural organic matter, light exposure, and transformation products [209].

Despite substantial progress, AI-assisted nanotoxicity prediction remains limited by data scarcity, endpoint inconsistency, and insufficient external validation. Toxicological datasets are often small, imbalanced, and biased toward positive findings. Negative or inconclusive toxicity results are rarely reported, limiting model robustness [210]. In addition, many models perform well on internal datasets but fail when applied to new materials, organisms, or exposure conditions. Future progress requires standardized reporting of nanomaterial characterization, exposure conditions, assay protocols, biological endpoints, and negative results. Safe-by-design antimicrobial nanomaterials will require integration of efficacy, toxicity, environmental fate, and regulatory considerations from the earliest stages of design.

### 5.4. Multimodal Data Integration and Explainable AI for Mechanistic Design

Although predictive accuracy is important, the long-term value of AI in antimicrobial nanomaterial design depends on whether models can provide a mechanistic understanding. Many early ML models functioned as black-box predictors, offering limited explanation of why a given nanomaterial was predicted to be antimicrobial or toxic. However, antimicrobial nanomaterials operate through multiple overlapping mechanisms, including membrane disruption, ROS generation, ion release, protein oxidation, DNA damage, enzyme inhibition, metabolic interference, and biofilm matrix disruption [31]. Therefore, predictive modeling must be connected to mechanistic interpretation if it is to support rational design.

Multimodal data integration provides a promising pathway toward mechanistic AI-guided design. Instead of relying only on tabular physicochemical descriptors, multimodal AI systems can integrate microscopy, spectroscopy, omics, microbiological assays, computational simulation, and literature-derived information [211]. For example, SEM and TEM images provide particle morphology and aggregation information; XRD reveals crystallinity and phase structure; FTIR and Raman spectroscopy identify chemical bonding and surface functional groups; ICP-MS quantifies ion release; DLS and zeta potential characterize colloidal behavior; and transcriptomics or proteomics can reveal microbial stress responses [212]. Combining these data types allows AI models to connect nanomaterial structure with antimicrobial mechanism more directly.

**Figure 5:**
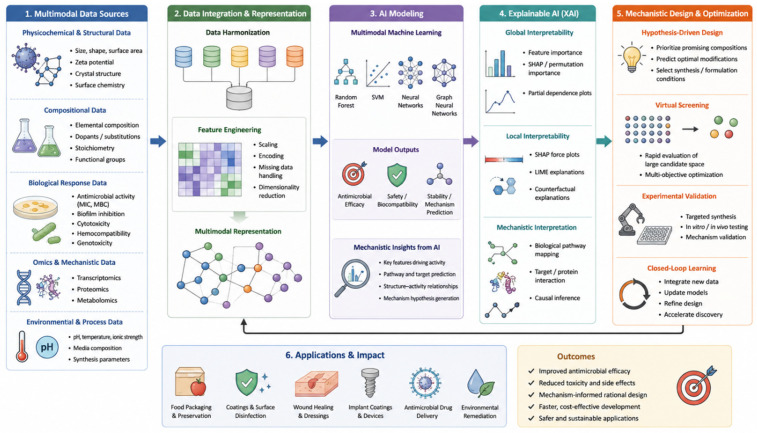
Multimodal data integration and explainable AI framework for mechanistic design of antimicrobial nanomaterials.

Figure 5 illustrates an integrated multimodal AI framework for mechanistic antimicrobial nanomaterial design, where diverse experimental, physicochemical, biological, and computational datasets are combined with explainable machine learning models to support predictive optimization and mechanistic interpretation. Deep learning methods are especially useful for multimodal analysis because they can process high-dimensional imaging and spectral datasets [213]. Convolutional neural networks can extract morphological features from microscopy images, while graph neural networks can represent atomic or molecular structures [84]. Transformer-based architectures may integrate textual, numerical, spectral, and image-based information into unified representations. This is important because manually selected descriptors may miss subtle but biologically important features, such as aggregation patterns, defect structures, surface roughness, or spectral signatures associated with reactive sites [214].

Explainable AI is essential for transforming predictive models into design tools. Feature-importance analysis, SHAP values, LIME, partial dependence plots, and counterfactual explanations can identify which variables drive antimicrobial predictions [41]. For antimicrobial nanomaterials, such tools may reveal whether bacterial inhibition is primarily associated with particle size, zeta potential, dopant content, band gap energy, ion release, or exposure time [215]. These insights can generate experimentally testable hypotheses and help researchers distinguish correlation from a plausible mechanism.

Mechanistic AI is particularly important for antibiofilm applications. Biofilms are structurally and metabolically heterogeneous communities embedded in extracellular polymeric substances. Their resistance to treatment arises from restricted diffusion, altered metabolism, persister cells, quorum sensing, and protective matrix components [216]. A model trained only on planktonic MIC data may fail to predict antibiofilm efficacy because biofilm inhibition depends on penetration, surface adhesion, matrix disruption, localized release, and sustained activity [217]. Future AI models should therefore include biofilm-specific descriptors such as matrix composition, biofilm thickness, bacterial community structure, surface roughness, quorum-sensing activity, and diffusion limitations.

For food systems, mechanistic AI models must account for matrix effects. Food proteins, carbohydrates, lipids, salts, organic matter, and pH can adsorb onto nanomaterial surfaces, alter aggregation, reduce ROS availability, bind released ions, or interfere with microbial contact [87]. For biomedical systems, protein corona formation can similarly change nanoparticle identity, cellular uptake, immune recognition, and antimicrobial performance [218]. Therefore, mechanistic models must incorporate the fact that nanomaterials do not behave identically in water, culture medium, food matrices, serum, wound exudate, or biofilm environments.

Another important opportunity involves integrating AI with molecular simulation. Density functional theory, molecular dynamics, and coarse-grained simulations can generate descriptors related to adsorption energy, membrane interaction, ion release tendency, ligand binding, or ROS generation potential [219]. These simulation-derived descriptors can help ML models connect atomic-scale structure with biological function. This is especially valuable for MOFs, doped metal oxides, and hybrid nanostructures, where antimicrobial performance may depend on subtle electronic or surface-level properties [220].

The future of data-driven antimicrobial nanomaterial design will likely involve integrated platforms that combine prediction, mechanism, safety, and application performance. These systems should not merely rank materials by predicted antimicrobial activity; they should explain why a material works, under which environmental conditions it remains effective, what mechanisms are likely involved, and where safety boundaries exist. Such integrated mechanistic AI frameworks will be essential for translating antimicrobial nanomaterials from laboratory discovery to food safety and biomedical implementation.

### 5.5. Toward Closed-Loop and Application-Specific Design Frameworks

The next stage of data-driven antimicrobial nanomaterial research will likely involve closed-loop and application-specific design frameworks. In these systems, AI models, automated synthesis, characterization tools, antimicrobial assays, and toxicity screening are integrated into iterative workflows [221]. Rather than testing a fixed set of formulations, the system learns from each experiment and proposes the next experiment most likely to improve performance. This approach is especially suitable for antimicrobial nanomaterials because the design space is large, the performance requirements are multidimensional, and biological responses are highly context-dependent.

A closed-loop antimicrobial nanomaterial development workflow may begin with literature-mined information and existing experimental datasets, from which machine learning models identify promising material compositions, surface modifications, and formulation conditions with high predicted antimicrobial potential [172]. Selected candidates can then be synthesized using automated platforms and systematically characterized through DLS, zeta potential analysis, spectroscopy, microscopy, and ion-release measurements to establish structure–property relationships [222]. Their antimicrobial performance may subsequently be evaluated using automated microplate assays, live/dead imaging, biofilm quantification, and parallel cytotoxicity or environmental safety assessment. The experimentally validated outputs are continuously reintegrated into the machine learning framework, enabling iterative refinement of predictive accuracy and guiding subsequent cycles of rational nanomaterial optimization.

Recent advances in autonomous synthesis platforms further support the feasibility of closed-loop antimicrobial nanomaterial development workflows. Park et al. demonstrated robotic and machine learning-enabled closed-loop optimization for nanoparticle synthesis, while Gao et al. developed an autonomous chemical platform integrating AI-guided decision-making with automated experimentation for end-to-end nanomaterial development [76,223]. In particular, Gao et al. emphasized the inefficiency and limited reproducibility associated with conventional trial-and-error synthesis approaches and proposed a data-driven framework capable of adaptive optimization through iterative experimental feedback. Collectively, these studies suggest that autonomous and AI-assisted nanomaterial development is progressively transitioning from conceptual design toward practical and scalable implementation.

For antimicrobial applications, however, closed-loop design must be adapted to biological complexity. The model should not optimize only particle size or optical response; it must optimize practical antimicrobial performance under relevant conditions. For food safety applications, this may include reduction of foodborne pathogens on produce surfaces, inhibition of biofilm formation on stainless steel, maintenance of food quality, low migration into food, and compatibility with regulatory requirements [206]. For biomedical applications, this may include bacterial killing in wound-like environments, antibiofilm activity on implants, low mammalian cytotoxicity, controlled degradation, and compatibility with sterilization or storage processes [146].

Application-specific modeling is essential because antimicrobial nanomaterials must be optimized according to their intended use environment. Materials effective for food-contact surfaces may not be suitable for wound healing, while formulations active against planktonic bacteria may fail in biofilms or complex food matrices [217]. Therefore, data-driven frameworks should define application-specific performance targets, such as migration stability for food packaging, tissue compatibility for wound dressings, and long-term antibiofilm protection for implant coatings [36].

Standardization will be critical for making closed-loop antimicrobial nanomaterial design reliable. Datasets should include complete metadata on synthesis conditions, characterization methods, microbial strains, assay protocols, exposure environments, and safety endpoints. Without standardized data, AI models may generate predictions that appear statistically accurate but lack practical transferability. FAIR data principles, open repositories, and harmonized assay reporting can substantially improve reproducibility and model generalization [224].

Data-driven design is not intended to replace experimental validation, but rather to enhance the efficiency, precision, and mechanistic depth of antimicrobial nanomaterial development. The most effective platforms will likely emerge from integrated workflows that combine computational prediction with systematic synthesis, physicochemical characterization, antimicrobial evaluation, toxicity assessment, and application-relevant validation. Through this integration, antimicrobial nanotechnology can progressively transition from a predominantly empirical field toward a more predictive, rational, and application-oriented discipline.

## 6. Applications in Food and Biomedical Systems

This section focuses on how antimicrobial nanomaterials are translated into food and biomedical platforms. In practical settings, performance depends not only on antimicrobial potency but also on matrix compatibility, safety, release stability, mechanical durability, sensory acceptability, regulatory feasibility, and real-world validation.

### 6.1. Food Preservation and Active Packaging

Food preservation and active packaging are among the most promising application areas for antimicrobial nanomaterials because microbial contamination can occur throughout production, handling, storage, distribution, and consumer use. Pathogens such as *Salmonella enterica*, *Escherichia coli* O157:H7, *Listeria monocytogenes*, *Staphylococcus aureus*, and spoilage microorganisms can persist on fresh produce, meat, seafood, dairy products, and food-contact materials [225]. In these systems, antimicrobial nanomaterials are not simply evaluated by their ability to inhibit bacteria in culture media; with the function within chemically complex food matrices containing proteins, lipids, salts, organic acids, carbohydrates, moisture gradients, and variable pH. These components can influence nanoparticle aggregation, migration, release behavior, and microbial contact, making application-specific evaluation essential.

Active packaging differs from conventional passive packaging because it interacts with the food or package headspace to delay deterioration, suppress microbial growth, reduce oxidation, or monitor quality changes. Nanomaterials can improve packaging performance by enhancing mechanical strength, reducing gas and moisture permeability, supporting sustained antimicrobial release, or introducing sensing functions [226]. Recent reviews have emphasized that nanomaterials-enabled active packaging can extend shelf life by delaying microbial spoilage and quality degradation, with examples including silver nanoparticles, ZnO, TiO_2_, nanocellulose, nanoclays, carbon-based nanomaterials, and biopolymer nanocomposites [227,228]. Nirmal et al. (2024) reviewed nanomaterial-enabled active food packaging and highlighted their role in delaying food deterioration and extending shelf life, while also identifying safety, cost, and scalability as remaining barriers [228].

Biopolymer-based packaging systems are especially relevant for sustainable food applications because they can combine biodegradability with antimicrobial performance. Chitosan, starch, gelatin, cellulose, polylactic acid, and protein-based matrices have been widely used to incorporate antimicrobial nanoparticles or nanoemulsions. Herrera-Rivera et al. (2024) reviewed the role of nanoparticles in food packaging materials and emphasized that nanostructures with high surface area and antimicrobial activity can be incorporated into biopolymer matrices to improve packaging functionality [229]. Similarly, Pattnaik et al. (2024) discussed nanotechnology-based food packaging and highlighted its advantages, while stressing that safety considerations and regulatory issues remain central for translation [230].

Specific examples include AgNP-containing films for inhibition of foodborne bacteria, ZnO-loaded packaging films for antimicrobial and UV-barrier functions, TiO_2_-based films for photocatalytic or self-cleaning activity, and essential oil nanoemulsion coatings for fresh produce and meat preservation [231]. In the essential oil nanoemulsion systems, encapsulation can improve dispersion of hydrophobic antimicrobials such as thymol, carvacrol, eugenol, cinnamaldehyde, oregano oil, and tea tree oil [232]. These systems may be particularly useful for minimally processed foods, where antimicrobial action must be achieved without harsh thermal treatment. However, sensory effects, volatility, oxidation, and interaction with food components remain important limitations.

Food-contact surfaces represent another important application area. Stainless steel, plastic, glass, and rubber surfaces in food-processing facilities can support microbial adhesion and biofilm formation. Antimicrobial nanocoatings may reduce contamination by limiting microbial attachment or providing localized antimicrobial release [233]. However, the most relevant evaluation criteria are not only planktonic bacterial killing but also coating durability, cleanability, resistance to abrasion, compatibility with sanitizers, and prevention of cross-contamination during repeated use [234]. AI-guided tools may support this field by modeling coating performance under different cleaning cycles, storage conditions, and food-contact scenarios.

Smart and AI-supported packaging is an emerging direction that connects antimicrobial nanomaterials with data analytics. AI-enabled packaging systems may integrate nanosensors, freshness indicators, temperature-abuse monitoring, and shelf-life prediction models [235]. Madhu et al. (2025) described AI-based food packaging systems as a frontier for sustainable and smart packaging, particularly through integration of sensing technologies and predictive analytics for quality monitoring [235]. In the context of antimicrobial nanomaterials, such systems could support real-time decisions on spoilage risk, microbial growth probability, and remaining shelf life.

Despite their considerable potential, antimicrobial nanomaterials for food applications continue to face multiple translational challenges, including nanoparticle migration into food matrices, toxicological uncertainty, consumer perception, labeling requirements, regulatory approval, and environmental persistence. Consequently, future research should emphasize realistic food matrix evaluation, migration and storage studies, sensory assessment, and life-cycle analysis to establish practical safety and performance beyond short-term antimicrobial testing.

### 6.2. Biomedical Infection Control and Wound Healing

Biomedical infection control is another major translational field for antimicrobial nanomaterials, particularly in wound healing, implant protection, catheter-associated infection control, topical therapy, and localized drug delivery [160]. Unlike food applications, biomedical platforms must function in dynamic biological environments containing proteins, enzymes, immune cells, tissue fluids, extracellular matrix components, and variable pH [172]. Therefore, application performance depends on more than antimicrobial potency. A clinically relevant antimicrobial nanomaterial must maintain activity under biological conditions while avoiding cytotoxicity, excessive inflammation, delayed tissue repair, hemolysis, immune overstimulation, or uncontrolled degradation [236].

Wound healing is one of the most active biomedical applications. Chronic wounds, diabetic ulcers, burn wounds, and surgical wounds are highly susceptible to microbial colonization and biofilm formation. Nanomaterial-based wound dressings can combine antimicrobial activity with moisture retention, exudate absorption, controlled release, and tissue-regenerative functions [237]. Nqoro et al. (2024) applied polymer-based wound dressings loaded with metal-based nanoparticles, including silver, gold, magnesium oxide, and zinc oxide, and emphasized their potential to provide antimicrobial and healing-supportive functions in wound environments [238]. Pino et al. (2023) developed nano-ZnO biocomposites for wound healing and described the development of films and hydrogels incorporating ZnO nanoparticles for antimicrobial wound-dressing platforms [239].

Silver-based wound materials remain among the most widely investigated antimicrobial nanoplatforms. AgNP-containing hydrogels, nanofibers, cotton fabrics, and polymer dressings have been studied for infected wounds because they provide broad antimicrobial activity and sustained release [240]. Kaya et al. (2025) determined recent advances of silver nanoparticles in wound healing and described AgNPs as contributing not only antibacterial activity but also anti-inflammatory, antioxidant, and cell-proliferation-related effects [241]. However, silver-based systems must be carefully optimized because excessive silver release can impair mammalian cell viability or delay tissue repair. This makes wound healing an ideal example where application-specific design must balance microbial inhibition with tissue compatibility.

ZnO-based wound systems also provide important examples. ZnO nanoparticles have been incorporated into biopolymer films, hydrogels, and composite dressings because of their antimicrobial activity, potential support of tissue repair, and relevance to skin-related applications [239]. Xiao et al. (2025) highlighted ZnO nanoparticles as promising for wound healing, particularly for mechanical injuries, diabetic ulcers, and burns [242]. Recent examples also include gelatin film dressings functionalized with silver and ZnO nanoparticles, where the combination of ZnO and AgNPs was described as enhancing antibacterial performance through intensified oxidative activity and membrane damage [243]. For this review, the key point is not to repeat the mechanism, but to show how these systems are being translated into dressings that require release control, flexibility, hydration, and cytocompatibility.

Medical implants and indwelling devices represent another important biomedical application. Orthopedic implants, dental implants, catheters, vascular grafts, and contact lenses are susceptible to bacterial adhesion and biofilm-associated infection [244]. Nanomaterial-based coatings may provide localized antimicrobial protection while avoiding systemic antibiotic exposure [245]. Sahoo et al. (2022) reviewed antimicrobial coatings for biomedical implants and devices, including metals, metal oxides, two-dimensional nanomaterials, and composite coatings [246]. Sui et al. (2024) reviewed nanomaterials for anti-infection in orthopedic implants and emphasized that nanomaterials can be used as both bactericidal surfaces and localized antimicrobial carriers [247]. Akay et al. (2024) further summarized advances in antibacterial coatings for orthopedic implants, highlighting the clinical relevance of preventing bacterial adhesion and colonization on implant surfaces [248].

Recent research has also explored copper oxide nanoparticle coatings for medical devices. A report described copper oxide nanoparticles developed as antipathogenic coatings for device materials, including silicone, stainless steel, and titanium, to reduce infection risk after implantation [249]. Such studies illustrate an important translational direction: antimicrobial nanomaterials must be compatible with clinically relevant device substrates and remain functional under sterilization, implantation, and physiological exposure conditions. Table 3 presents representative biomedical applications of antimicrobial nanomaterials, linking each platform to its translational function and the key technical barriers that must be addressed for clinical implementation.

Drug-delivery applications provide another biomedical pathway. Polymeric nanoparticles, liposomes, nanogels, MOFs, and hybrid nanocarriers can deliver antibiotics, antimicrobial peptides, photosensitizers, quorum-sensing inhibitors, or natural antimicrobials to infected tissues or biofilms [251]. These systems are particularly relevant where conventional antibiotics fail due to poor penetration, rapid clearance, or systemic toxicity. However, biomedical translation requires demonstration of pharmacokinetics, biodistribution, degradation behavior, immune compatibility, and therapeutic benefit in appropriate infection models.

To sum up, biomedical applications require stricter validation than many food applications because materials may contact living tissue directly. Future studies should include not only bacterial inhibition but also mammalian cell compatibility, wound closure, inflammatory markers, hemocompatibility, sterilization stability, and in vivo infection outcomes. AI-guided frameworks may support these efforts by integrating antimicrobial efficacy, release kinetics, cytotoxicity, and tissue-response data, but clinical translation will require robust experimental validation.

### 6.3. Antimicrobial Surfaces and Biofilm-Prone Interfaces

Antimicrobial surfaces and biofilm-prone interfaces represent a translational bridge between food and biomedical systems. In both environments, microbial adhesion to surfaces can initiate persistent contamination and biofilm formation [252]. In food-processing facilities, biofilms may develop on stainless steel, conveyor belts, cutting equipment, plastic surfaces, rubber seals, and packaging-contact materials [171]. In healthcare environments, biofilms can form on implants, catheters, wound dressings, dental materials, and hospital surfaces [244]. Because biofilms are more difficult to remove than planktonic cells, practical antimicrobial platforms must be designed not only to inhibit microbes but also to prevent adhesion, withstand cleaning or physiological exposure, and maintain long-term activity.

**Figure 6:**
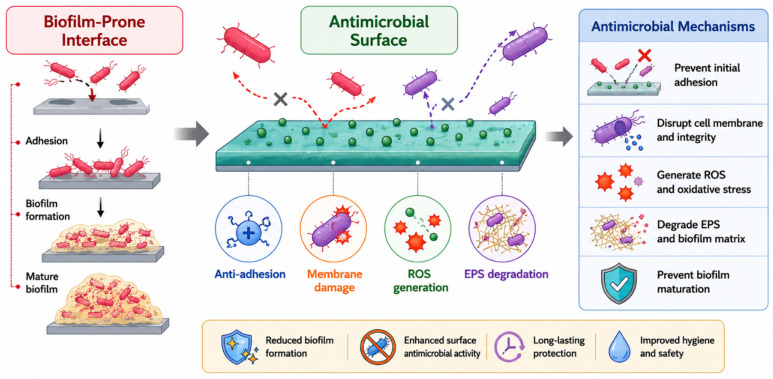
Design Strategies for Antimicrobial and Antibiofilm Surfaces.

Figure 6 illustrates representative biofilm-prone environments, including medical devices, water systems, food-processing surfaces, and dental interfaces, together with major antimicrobial surface strategies such as nanoparticle coatings, surface functionalization, topographical engineering, stimuli-responsive materials, and self-cleaning surfaces.

For food-processing surfaces, nanomaterial coatings may be applied to stainless steel or polymeric materials to reduce microbial attachment and improve cleanability [114,115]. The most important design requirements include coating adhesion, abrasion resistance, sanitizer compatibility, low migration, non-corrosiveness, and maintenance of antimicrobial performance after repeated cleaning [253]. These performance criteria distinguish surface applications from suspension-based antimicrobial tests.

For biomedical surfaces, the criteria differ. Implant and device coatings must remain stable under physiological conditions, resist protein fouling, prevent bacterial colonization, and maintain compatibility with host cells. Antimicrobial coatings for orthopedic and dental implants may incorporate silver, copper, ZnO, TiO_2_, hydroxyapatite, graphene derivatives, antimicrobial peptides, antibiotics, or polymeric nanostructures [254]. Negut et al. (2024) reviewed antimicrobial coatings for dental implants and noted the importance of coating strategies in reducing implant-associated infection risks [255]. These examples demonstrate that surface antimicrobial systems must be engineered for both microbiological and mechanical performance.

Antibiofilm food packaging is also emerging as a specialized application. A review on nanoparticle-based antibiofilm food packaging materials emphasized the need to consolidate recent research on packaging systems designed specifically to prevent biofilm formation during long-term storage [113]. Future packaging systems should therefore include biofilm-specific endpoints such as biomass reduction, live/dead imaging, extracellular polymeric substance disruption, and surface recolonization after storage.

Nanomaterial-enabled surface decontamination also extends to water systems and engineered environments. Cao et al. (2024) reviewed advances in applying nanomaterials for biofilm control and emphasized that nanomaterials can be used across water-treatment, healthcare, and industrial systems where biofilm formation causes persistent operational and safety problems [256]. Design principles, such as surface durability, antifouling behavior, controlled antimicrobial release, and environmental safety, also apply to food and biomedical interfaces.

AI-guided analysis can support antimicrobial surface development by connecting surface properties with biofilm outcomes. For example, models may incorporate surface roughness, hydrophobicity, coating thickness, release rate, microbial species, cleaning frequency, and exposure environment to predict long-term antimicrobial performance. In food-processing applications, this could support risk-based sanitation planning [257]. Within biomedical applications, it could help identify coating designs that minimize bacterial adhesion while preserving host cell integration.

Antimicrobial surface design should move beyond short-term inhibition assays toward long-term performance evaluation under practical conditions. Food surfaces should be evaluated under repeated cleaning, organic loading, temperature fluctuation, and contact with real food residues. Biomedical surfaces should be tested under protein-rich fluids, mechanical stress, sterilization, and cell–material interaction models [248,258]. Without these conditions, promising laboratory results may overestimate translational performance.

### 6.4. Application-Specific Design Criteria and Translational Barriers

The translation of antimicrobial nanomaterials into food and biomedical systems requires application-specific design criteria. Although both fields seek microbial control, their performance requirements differ substantially. Food applications prioritize shelf-life extension, pathogen reduction, packaging compatibility, sensory preservation, migration control, regulatory compliance, and consumer acceptance [206]. Biomedical applications prioritize cytocompatibility, hemocompatibility, tissue integration, immune response, sterilization stability, degradation behavior, and clinical efficacy [259]. Therefore, a nanomaterial that performs well in one context may not be appropriate for another.

In food systems, migration is one of the most important translational concerns. Nanoparticles or released ions may migrate from packaging into food, especially under high temperature, high fat content, acidic conditions, or prolonged storage. This makes migration testing essential for active packaging and edible-coating applications. Sensory effects are also important because antimicrobial compounds such as essential oils may alter aroma, taste, or appearance [260]. Regulatory approval depends on material identity, exposure route, migration level, toxicological evidence, and intended food-contact use. Therefore, food-directed antimicrobial nanomaterials must be optimized not only for bacterial inhibition but also for migration safety, food quality, and consumer acceptability.

In biomedical systems, direct contact with tissue imposes a different set of requirements. Wound dressings must maintain moisture balance, absorb exudate, release antimicrobials at appropriate rates, support tissue repair, and avoid excessive cytotoxicity [261]. Implant coatings must adhere strongly to substrates, resist mechanical wear, remain stable during sterilization, and prevent bacterial colonization without impairing osseointegration or tissue compatibility. Drug-delivery systems must demonstrate appropriate pharmacokinetics, biodistribution, degradation, and immune safety. These requirements are more complex than antimicrobial potency alone [262].

Manufacturing and scalability are also major barriers. Many antimicrobial nanomaterials are synthesized under laboratory conditions that are difficult to scale reproducibly. Batch-to-batch variation in particle size, surface charge, release behavior, and antimicrobial activity can limit industrial or clinical translation [263]. For packaging applications, materials must be compatible with extrusion, casting, coating, lamination, or printing processes [264]. Similarly, for biomedical applications, they must be compatible with sterilization, storage, quality-control standards, and regulatory manufacturing requirements.

Environmental sustainability should also be incorporated into translational evaluation. Antimicrobial nanomaterials may enter waste streams through packaging disposal, washing, wound dressing disposal, or medical-device waste. Metal-based nanoparticles may persist or transform in environmental systems, affecting microbial communities or aquatic organisms [265]. Therefore, life-cycle assessment and environmental fate studies should become part of responsible material development, especially for large-volume food packaging applications.

AI-guided design offers a promising strategy for addressing these translational challenges by integrating multiple performance criteria within multi-objective optimization frameworks. In food-related applications, predictive models can simultaneously evaluate antimicrobial efficacy, migration behavior, shelf-life extension, packaging integrity, and sensory quality, whereas biomedical models may incorporate bacterial inhibition, release kinetics, mammalian cell compatibility, inflammatory response, and coating stability [266]. Nevertheless, the reliability and practical applicability of these models ultimately depend on the availability of standardized, high-quality datasets generated under realistic application conditions.

## 7. Conclusions

The convergence of data-driven design and antimicrobial nanomaterials is reshaping how functional nanosystems are discovered, optimized, and translated for food and biomedical applications. Rather than relying solely on empirical synthesis and endpoint antimicrobial testing, the field is moving toward a more predictive framework in which material composition, physicochemical descriptors, biological interactions, and application conditions can be systematically connected. This transition is particularly important for antimicrobial nanomaterials because their performance depends on multiple interrelated factors, including particle size, morphology, surface charge, dopant chemistry, release behavior, matrix compatibility, and microbial phenotype.

Antimicrobial nanomaterials, including metal and metal oxide nanoparticles, metal–organic frameworks, polymeric nanocarriers, nanoemulsions, and hybrid platforms, offer considerable promise for controlling microbial contamination, suppressing biofilm formation, and addressing antimicrobial resistance. Their value lies not only in their ability to damage microbial cells, but also in their tunability for controlled release, surface modification, targeted delivery, and multifunctional integration. These features make them relevant to active food packaging, edible coatings, food-contact surfaces, wound dressings, implant coatings, localized drug delivery, and antibiofilm systems. However, strong antimicrobial activity alone is not sufficient for practical implementation. Translation requires careful consideration of cytocompatibility, migration behavior, environmental fate, storage stability, manufacturability, regulatory acceptance, and performance under realistic food or biological conditions.

Artificial intelligence and machine learning provide important tools for addressing these challenges. Descriptor-based modeling can help identify relationships between nanomaterial properties and antimicrobial outcomes, while ML-guided synthesis and formulation strategies can reduce experimental burden and improve reproducibility. AI-assisted nanotoxicity prediction further supports safe-by-design development by allowing safety considerations to be incorporated earlier in the material design process. In addition, explainable AI and multimodal data integration offer opportunities to move beyond black-box prediction toward mechanistic interpretation, which is essential for scientific credibility and regulatory confidence.

Despite these advances, several barriers continue to limit the maturity of AI-guided antimicrobial nanotechnology. Current datasets remain fragmented, heterogeneous, and often incomplete, with substantial variation in synthesis protocols, characterization methods, microbial assays, exposure conditions, and endpoint reporting. Many studies still evaluate antimicrobial activity under simplified in vitro conditions, which may not reflect the complexity of food matrices, biofilms, wound exudates, physiological fluids, or long-term storage environments. Limited external validation and insufficient reporting of negative or unsuccessful results further restrict model generalizability. These issues must be addressed before data-driven models can reliably guide material selection and application-specific design.

Future progress will depend on stronger integration among nanomaterial engineering, microbiology, toxicology, food science, biomedical science, and data science. Standardized datasets, harmonized antimicrobial testing protocols, data practices, and transparent model reporting will be essential for improving reproducibility and cross-study comparison. Closed-loop platforms that combine high-throughput synthesis, automated characterization, biological screening, and explainable ML have the potential to accelerate the development of antimicrobial nanomaterials with balanced efficacy and safety. At the same time, application-specific validation should remain central, because materials designed for food packaging, wound healing, or implant protection face distinct performance requirements and regulatory pathways.

Data-driven antimicrobial nanomaterial design therefore represents more than a computational enhancement of existing workflows. It offers a framework for developing more predictive, safer, and application-relevant antimicrobial systems. Continued progress will depend on rigorous validation, transparent reporting, and responsible development to support next-generation solutions for food safety, infection control, and biofilm-associated challenges in both industrial and clinical settings.

## Figures and Tables

**Table 1:** Polymeric nanocarriers and nanoemulsion-based antimicrobial systems for food and biomedical applications [131,132,133,134,135,136,137,138,139,140].

Nanomaterial System	Antimicrobial Function and Advantages	Applications and Limitations
Chitosan-based nanoparticles	Positively charged chitosan interacts with negatively charged microbial membranes, disrupting them and causing leakage of intracellular contents. It also serves as a biodegradable carrier for essential oils, antibiotics, phenolics, and metal nanoparticles [131].	Used in edible coatings, antimicrobial packaging, food-contact surfaces, wound dressings, and drug delivery. Limitations include pH-dependent solubility, aggregation, and variable stability in complex food or biological matrices [132].
Protein- and polysaccharide-based nanocarriers	Alginate, gelatin, whey protein, soy protein, and related biopolymers protect antimicrobial compounds, improve dispersion, and enable controlled release [133]. These systems are attractive because they are generally biodegradable and suitable for food-grade delivery.	Applied in food preservation, edible coatings, active packaging, oral delivery, and wound-healing matrices. Limitations include weak intrinsic antimicrobial activity and sensitivity to pH, enzymes, ionic strength, and processing conditions [134].
Essential oil nanoemulsions	Nanoemulsions improve the aqueous dispersion, stability, and antimicrobial contact of hydrophobic compounds such as thymol, carvacrol, eugenol, cinnamaldehyde, oregano oil, and tea tree oil. Their main mechanisms involve membrane disruption, enzyme inhibition, and leakage of cellular components [135].	Used in fresh-produce preservation, edible coatings, food washing, active packaging, and topical antimicrobial systems. Limitations include volatility, oxidation, sensory effects, surfactant selection, and storage instability [136].
Lipid nanocarriers and hydrogel–nanoparticle systems	Liposomes, solid lipid nanoparticles, nanostructured lipid carriers, nanogels, and hydrogel systems support sustained antimicrobial release, localized delivery, moisture retention, and antibiofilm activity [137].	Mainly applied in wound dressings, burn treatment, implant-associated infection control, topical antimicrobial therapy, and drug delivery. Limitations include oxidative instability, payload leakage, mechanical weakness, sterilization challenges, and scale-up difficulty [138].
Polymer–metal and AI-optimized hybrid systems	Hybrid systems combine polymer-mediated adhesion or controlled release with metal ion release, ROS generation, or contact-mediated killing. AI and ML can optimize particle size, charge, encapsulation efficiency, release kinetics, antimicrobial activity, and cytotoxicity [139].	Promising for antimicrobial packaging, food-contact coatings, wound dressings, biomedical coatings, and smart delivery systems. Limitations include possible metal toxicity, migration concerns, complex regulatory evaluation, and the need for high-quality datasets and model validation [140].

**Table 2:** Machine learning-guided synthesis and formulation optimization of antimicrobial nanomaterials [188,192,196,197,198].

Optimization Strategy	Target Parameters and Model Outputs	Relevance to Antimicrobial Nanomaterial Design
Bayesian optimization	Optimize synthesis variables such as precursor concentration, pH, temperature, reaction time, reducing agent, and stabilizer concentration. Outputs may include particle size, morphology, optical response, yield, and stability.	Particularly useful when experimental datasets are small, and each experiment is time-consuming. It can identify optimal synthesis conditions with fewer trials and support efficient development of AgNPs, ZnO NPs, MOFs, and hybrid antimicrobial systems [196].
High-throughput screening combined with ML	Screens large formulation libraries using automated synthesis, microfluidics, or robotic platforms. Outputs include particle size, polydispersity, zeta potential, encapsulation efficiency, antimicrobial activity, and cytotoxicity.	Enables rapid comparison of multiple nanomaterial formulations and helps identify candidates with balanced antimicrobial efficacy, stability, and safety. This is valuable for food packaging, produce washing, wound dressings, and implant coatings [197].
Multi-objective optimization	Simultaneously optimizes multiple outcomes, including antimicrobial activity, antibiofilm performance, mammalian cell viability, controlled release, colloidal stability, scalability, and cost.	Prevents over-optimization of antimicrobial potency alone and supports safer, application-specific nanomaterial design, with higher ion release or ROS generation may improve bacterial killing but also increase cytotoxicity [198].
ML-guided nanoemulsion and polymeric formulation design	Predicts formulation-dependent properties such as droplet size, encapsulation efficiency, release kinetics, storage stability, and antimicrobial activity based on polymer ratio, oil phase, surfactant type, homogenization conditions, and ionic strength.	Useful for optimizing essential oil nanoemulsions, chitosan nanoparticles, PLGA carriers, and antimicrobial hydrogels. These systems are relevant for edible coatings, active packaging, topical antimicrobials, and wound-healing platforms [192].
Closed-loop autonomous optimization	Integrates AI prediction, automated synthesis, characterization, biological testing, and feedback learning. Outputs are continuously updated to guide the next experimental cycle.	Provides a future-oriented framework for self-optimizing antimicrobial nanomaterials. It can accelerate discovery, improve reproducibility, and support application-specific design under realistic food and biomedical conditions [188].

**Table 3:** Biomedical and clinical applications of antimicrobial nanomaterials [152,154,237,246,250].

Biomedical Application	Representative Antimicrobial Nanomaterial Systems	Translational Relevance and Key Limitations
Wound dressings and chronic wound care	AgNP-loaded hydrogels, ZnO-based films, chitosan nanoparticles, polymer–metal nanocomposites, nanoemulsion-loaded dressings	Support localized antimicrobial activity, moisture retention, sustained release, and reduced wound bioburden. Major limitations include cytotoxicity at high doses, burst release, sterilization stability, and the need to demonstrate wound closure and tissue compatibility in realistic models [237].
Implant and medical-device coatings	AgNP, CuO, ZnO, TiO_2_, graphene-based coatings, MOF-based coatings, polymer–nanoparticle composite coatings	Used to reduce bacterial adhesion and biofilm formation on orthopedic implants, dental implants, catheters, and device surfaces. Key challenges include coating durability, mechanical stability, protein fouling, long-term release control, and compatibility with host cell integration [246].
Localized antimicrobial drug delivery	PLGA nanoparticles, liposomes, nanogels, dendrimers, MOFs, lipid nanoparticles loaded with antibiotics or antimicrobial peptides	Improve local drug concentration, reduce systemic toxicity, and enable sustained or stimuli-responsive release. Limitations include variable biodistribution, premature payload leakage, immune clearance, degradation behavior, and scale-up complexity [154].
Antibiofilm therapy	Nanoemulsions, polymeric nanoparticles, AgNPs, ZnO NPs, photothermal nanoparticles, photodynamic nanoplatforms	Designed to improve penetration into biofilm matrices, disrupt extracellular polymeric substances, and enhance antimicrobial susceptibility. Limitations include poor translation from planktonic assays, limited penetration in mature biofilms, and insufficient validation under clinically relevant biofilm conditions [152].
Infection-responsive and personalized treatment platforms	pH-responsive nanocarriers, enzyme-responsive hydrogels, AI-optimized nanomedicine systems, smart antimicrobial coatings	Enable application-specific or patient-specific antimicrobial delivery based on wound pH, bacterial enzymes, inflammation status, or infection severity. Challenges include complex formulation design, limited clinical data, regulatory uncertainty, and the need for interpretable AI-supported decision frameworks [250].

## Data Availability

No new data were created or analyzed in this study. Data sharing is not applicable to this article.
